# DDX3 localizes to the centrosome and prevents multipolar mitosis by epigenetically and translationally modulating p53 expression

**DOI:** 10.1038/s41598-017-09779-w

**Published:** 2017-08-25

**Authors:** Wei-Ju Chen, Wei-Ting Wang, Tsung-Yuan Tsai, Hao-Kang Li, Yan-Hwa Wu Lee

**Affiliations:** 1 Institute of Biochemistry and Molecular Biology, School of Life Sciences, National Yang-Ming University, Taipei, Taiwan; 2 Department of Biological Science and Technology, College of Biological Science and Technology, National Chiao-Tung University, Hsinchu, Taiwan

## Abstract

The DEAD-box RNA helicase DDX3 plays divergent roles in tumorigenesis, however, its function in mitosis is unclear. Immunofluorescence indicated that DDX3 localized to centrosome throughout the cell cycle and colocalized with centrosome-associated p53 during mitosis in HCT116 and U2OS cells. DDX3 depletion promoted chromosome misalignment, segregation defects and multipolar mitosis, eventually leading to G2/M delay and cell death. DDX3 prevented multipolar mitosis by inactivation and coalescence of supernumerary centrosomes. DDX3 silencing suppressed Ser^15^ phosphorylation of p53 which is required for p53 centrosomal localization. Additionally, knockout of p53 dramatically diminished the association of DDX3 with centrosome, which was rescued by overexpression of the centrosomal targeting-defective p53 S15A mutant, indicating that centrosomal localization of DDX3 is p53 dependent but not through centrosomal location of p53. Furthermore, DDX3 knockdown suppressed *p53* transcription through activation of DNA methyltransferases (DNMTs) along with hypermethylation of *p53* promoter and promoting the binding of repressive histone marks to *p53* promoter. Moreover, DDX3 modulated *p53* mRNA translation. Taken together, our study suggests that DDX3 regulates epigenetic transcriptional and translational activation of p53 and colocalizes with p53 at centrosome during mitosis to ensure proper mitotic progression and genome stability, which supports the tumor-suppressive role of DDX3.

## Introduction

Centrosome amplification and aneuploidy are hallmarks of cancer cells. In general, each cell has a single centrosome which duplicates once in S phase. During mitosis, the duplicated centrosomes separate and form the two poles of the mitotic spindle. Chromosomes are then captured by the mitotic spindles and equally segregated into two daughter cells^1^. Centrosome over-duplication or cytokinesis failure results in supernumerary centrosomes. By clustering or inactivating the excess centrosomes, cells with multiple copies of centrosomes satisfy pseudo-bipolar mitosis and exhibit mild aneuploidy. Otherwise, cells undergo multipolar mitosis, which leads to severe aneuploidy and poor survival^2, 3^. Survival of very few daughter cells that obtain an appropriate chromosome complement thereby contribute to clonal evolution of aneuploid cancer cells, which is linked to progressive development of invasive high-grade tumors^4, 5^. Therefore, the proper control of centrosome number and activity is essential for promoting faithful chromosome inheritance and genome stability^6^.

P53, a well-known tumor suppressor gene, is critical for centrosome duplication and regulation. Phosphorylation of p53 at serine 15 directs p53 to centrosome where p53 exerts mitotic checkpoint surveillance during mitosis. Serine 15 phosphorylation is essential for centrosomal p53-mediated mitotic checkpoint surveillance during mitosis^7, 8^. The centrosomally localized p53 also participates in the regulation of centrosome duplication in addition to its transactivation-dependent regulation^9^. Loss of p53 causes centrosome amplification which results in multiple mitotic spindle poles and aberrant chromosome segregation^10^. Moreover, in cleavage failure and centrosome over-duplicated tetraploid cells, p53 abnormality impairs clustering of centrosomes and causes multipolar mitosis along with a high degree of aneuploidy^11–13^. Therefore, p53 acts as the guardian of the genome by regulating centrosome for accurate mitotic progression and actively preserving genome stability.

The expression of p53 is tightly controlled through a variety of mechanisms, including transcriptional, epigenetic and translational regulations^14^. The *p53* promoter is regulated by the interplay of a number of transcription factors, including p53 itself^15^. Moreover, *p53* promoter has a CTCF binding site which serves as a barrier against the binding of repressive histone marks, such as H3K9me3, H4K20me3 and H3K27me3^16, 17^. Furthermore, by promoting auto-PARylation of PARP1 which in turn inhibits the DNA methyltransferase activity of DNMT1 via the ADP-ribose polymers, CTCF preserves the methylation-free status of CTCF-target sites^18^. The de novo DNA methyltransferase 3 A and 3B also participate in *p53* gene regulation. DNMT3A suppresses the transcription of p53-target genes through interaction with p53^19^, while DNMT3B has been reported to mediate *p53* DNA methylation^20, 21^. The *p53* mRNA contains internal ribosome entry site (IRES) in the 5′UTR. The 3′UTR base pairs with the 5′UTR to form a steady RNA structure that is crucial for translational regulation of *p53* mRNA^22–24^.

The DEAD-box RNA helicase DDX3 is involved in multiple biological pathways including immune response, viral replication, gene regulation and tumorigenesis^25, 26^. However, the role of DDX3 in tumorigenesis is controversial^27^. Interestingly, DDX3 positively or negatively regulates cell cycle progression and cell motility in a cell-type-specific manner^28–36^. Several studies indicate that low expression of DDX3 is closely related to tumor malignancy and poor clinical outcomes^30–32, 35, 36^, suggesting a tumor suppressor role of DDX3. Notably, DDX3 interacts with p53 and stimulates p53 accumulation^37^. Additionally, p53 positively regulates DDX3^36^. The interplay between DDX3 and the tumor suppressor p53 also supports the tumor suppressive role of DDX3. Moreover, DDX3 is crucial for cell cycle G2/M progression in *Drosophila* ^38^. Loss of DDX3 causes severe DNA damage along with cell growth retardation and also promotes apoptosis during mouse development^39, 40^, inferring that DDX3 plays a role in mitosis and is essential for mitotic genome stability. Coincidently, DDX3 has been reported to promote chromosome condensation for accurate chromosome segregation during anaphase^41^.

In this study, we found that DDX3 localized to centrosome and colocalized with centrosome-associated p53 during mitosis in *p53* wild-type HCT116 and U2OS cells. DDX3 knockdown suppressed Ser^15^ phosphorylation of p53 and centrosomal targeting of p53. p53 itself is also required for centrosomal targeting of DDX3, which is independent of the centrosomal localization of p53. Depletion of DDX3 caused high incidence of multipolar mitosis by impaired clustering and inactivation of extra centrosomes. Reintroduction of DDX3 reduced multipolar mitosis and cell death in the DDX3-knockdown cells. Furthermore, DDX3 positively modulated p53 expression epigenetically and translationally. Our results demonstrate that DDX3 controls centrosome activity for accurate mitotic progression and the maintenance of genome stability through regulation of p53, thus providing new insight into the tumor-suppressive activity of DDX3.

## Results

### DDX3 localizes to the centrosome throughout the cell cycle and colocalizes with centrosome-associated p53 during anaphase and telophase

**Figure 1:**
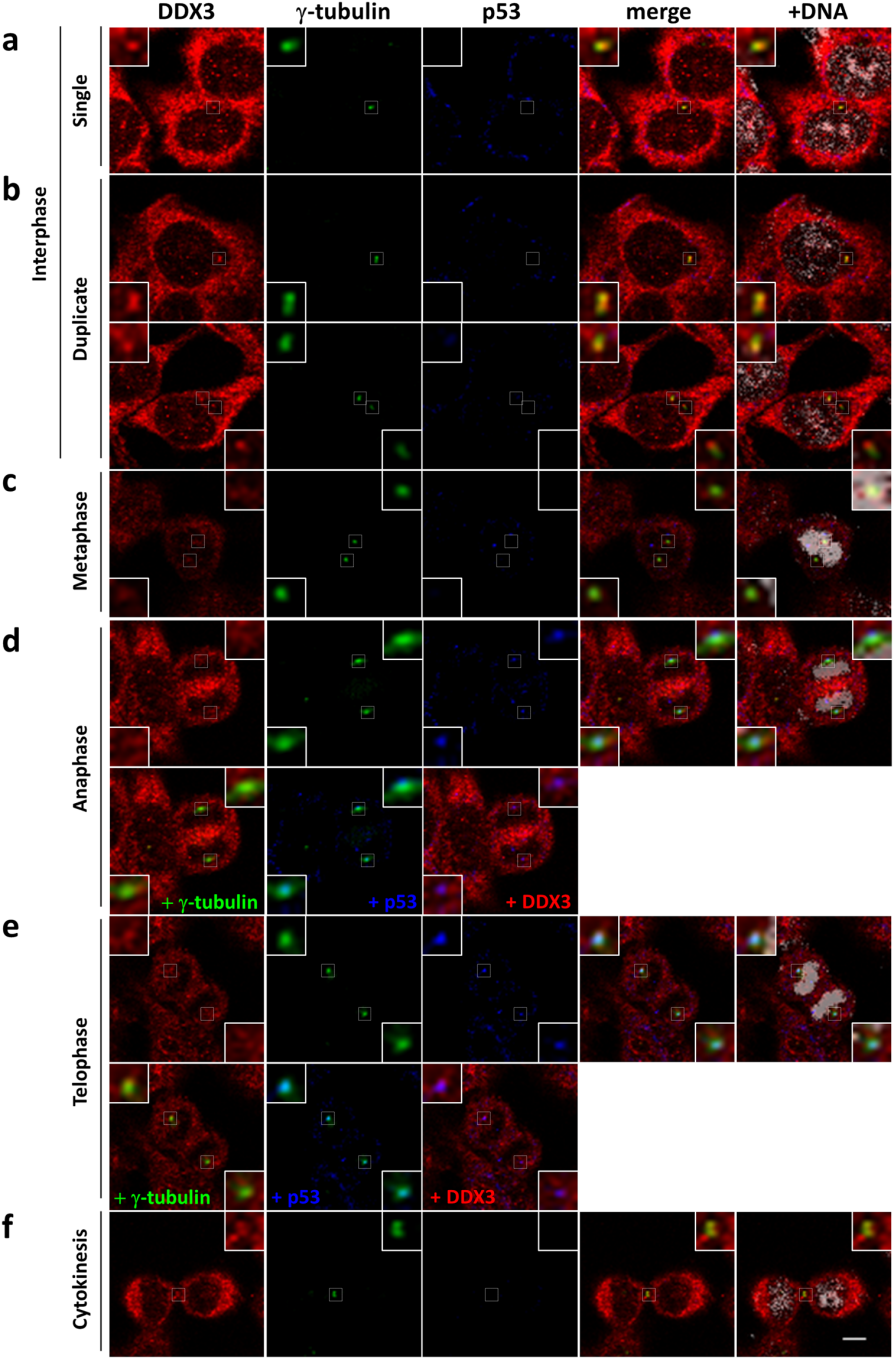
DDX3 localizes to the centrosome throughout the cell cycle and associates with p53 at centrosome during anaphase and telophase. HCT116 cells were immunostained with anti-DDX3 (red), anti-γ-tubulin (green), anti-p53 (blue) antibodies and DAPI (gray) during interphase (**a**-single centrosome; **b**-duplicated centrosomes), mitosis (**c**-metaphase; **d**-anaphase; **e**-telophase) and cytokinesis (**f**). Insets show higher magnifications of the centrosome (interphase, mitosis) and midbody (cytokinesis), respectively. Representative confocal images show colocalization of DDX3 with the centrosome in interphase and mitotic cells, and association of p53 with DDX3 and centrosome at anaphase and telophase. Additionally, DDX3 is concentrated at the midbody during cytokinesis. Scale bar = 5 μm.

HCT116 is a *p53* wild-type human colorectal cancer cell line that exhibits a near diploid phenotype^42^. Centrosome is the key regulator of mitosis and γ-tubulin is a centrosome marker. To explore the role of DDX3 in mitosis, we first investigated the subcellular localization of DDX3 through the cell cycle in HCT116 cells by immunofluorescence analysis with anti-DDX3 and anti-γ-tubulin antibodies. In interphase cells, DDX3 was concentrated and closely associated with both the single and newly duplicated centrosomes (Fig. 1a,b), while located primarily at the proximal end of the separate duplicated centrosomes. Notably, DDX3 was associated with the mitotic centrosomes and spread around the centrosome during metaphase, anaphase and telophase (Fig. 1c–e). Furthermore, in view of the centrosomal localization of p53 during mitosis^7, 8^ and that DDX3 interacts with p53^37^, we examined if DDX3 colocalizes with p53 at centrosome by immunofluorescence with anti-p53 antibody. DDX3 associated with p53 at centrosome during anaphase (Fig. 1d) and telophase (Fig. 1e). In cytokinesis, γ-tubulin has been reported to participate in the formation of midbody^43, 44^. We also found that DDX3 was condensed and overlapped with γ-tubulin at the midbody during cytokinesis (Fig. 1f). These observations demonstrate that DDX3 localizes to centrosome throughout the cell cycle and associates with p53 at centrosome during mitosis. Similar conclusion was also obtained in another *p53* wild-type U2OS cells (Supplementary Fig. S1).

### Downregulation of DDX3 results in mitotic abnormalities, G2/M phase transition delay and increased cell death

**Figure 2:**
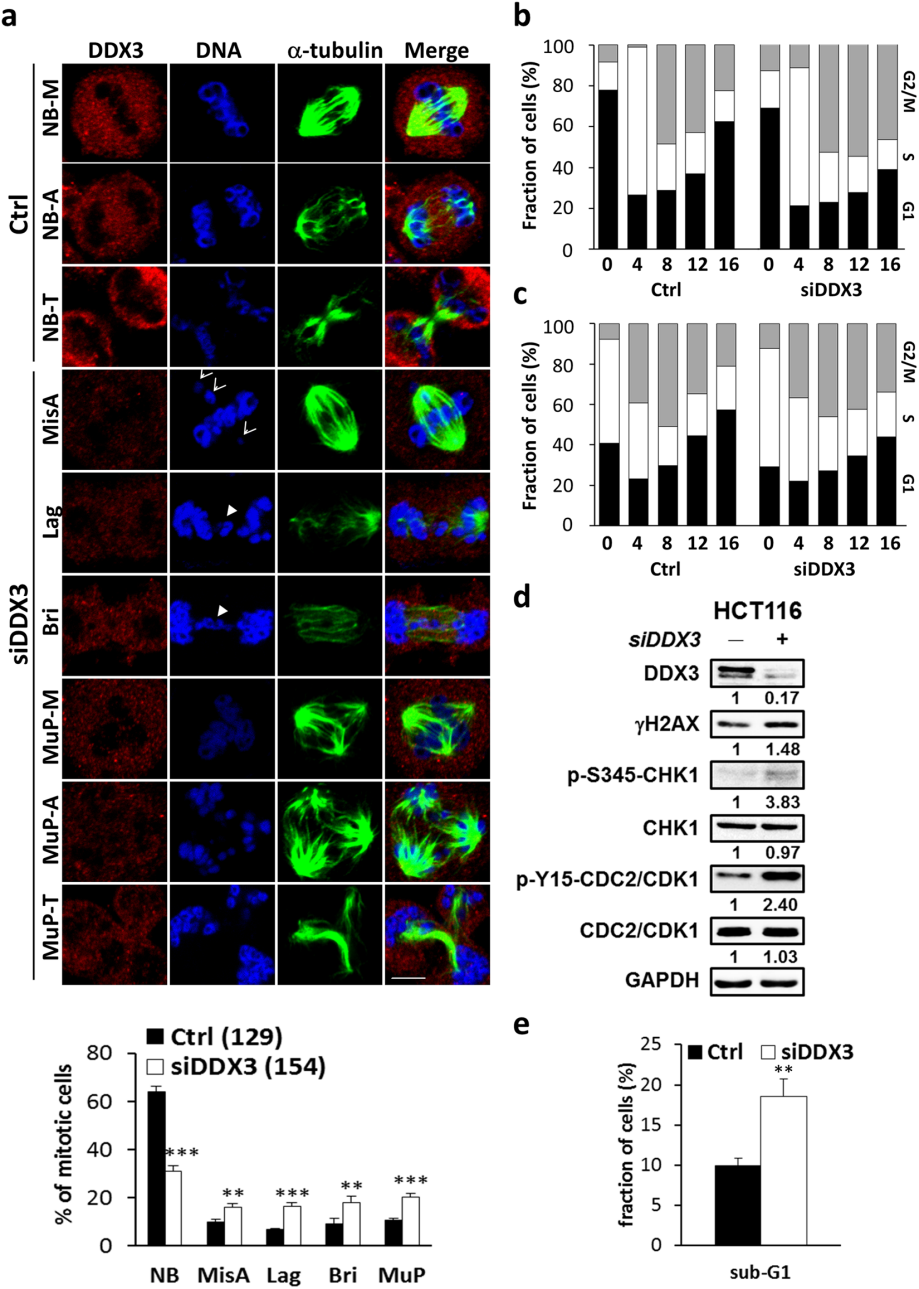
Downregulation of DDX3 results in mitotic abnormalities, G2/M phase transition delay and increased cell death. (**a**) Mitotic-enriched cells were immunostained with anti-DDX3 (red), anti-α-tubulin (green) antibodies and DAPI (blue). Representative confocal images show the control mitotic cells with normal bipolar spindles (NB) and balanced chromosome segregation in metaphase (NB-M), anaphase (NB-A) and telophase (NB-T) while DDX3 knockdown promoted the abnormal mitosis such as chromosome misalignment (MisA) (arrow), chromosome segregation defect (arrowhead), lagging chromosome (Lag) and chromosome bridge (Bri) and multipolar mitosis (MuP-M, A, T). Scale bar = 5 μm. The percentage of aberrant mitosis in the control and DDX3-knockdown cells were analyzed. Data are shown as the average value ± S.D. calculated from three independent experiments. **P < 0.01; ***P < 0.001. (n), the number of cells analyzed. (**b**) (**c**). Histogram of cell cycle phase distribution in the control and DDX3-knockdown cells. DDX3 knockdown induced prolonged accumulation in G2/M phase at 12–16 hr after release from G1 (**b**) or S phase block (**c**). Cells were synchronized at G1 (**b**) or S (**c**) phase at 24 hr post-transfection and harvested at indicated time points after release. Harvested cells were stained with propidium iodide and analyzed by flow cytometry. Results are representative data of two independent experiments. (**d**) Western blot analysis showing the expression of γH2AX, Ser^345^-phosphorylated CHK1, CHK1 kinase and Tyr^15^-phosphorylated CDK1 in the control and DDX3-knockdown cells. Depletion of DDX3 caused an increase of γH2AX, a double strand DNA break marker, and activation of CHK1 kinase, which led to accumulation of phosphorylated CDK1 and prevented G2/M transition. Original images of western blots were presented in Supplementary Fig. S3. (**e**) DDX3 knockdown increased the distribution of cells in the sub-G1 peak. The proportions of sub-G1 phase population of the control and DDX3-knockdown cells are shown as the average value ± S.D. calculated from three independent experiments. **P < 0.01.

To further examine the function of DDX3 in mitotic progression, control and DDX3-knockdown HCT116 cells were synchronized at mitosis and analyzed by immunofluorescence with anti-α-tubulin, the mitotic spindle marker and anti-DDX3 antibodies. Depletion of DDX3 caused an increase in aberrant chromosome segregation such as lagging and chromosome bridge from 6.9% to 16.4% and 9% to 17.8%, respectively, as compared to the control (Fig. 2a), which is consistent with the previous study^41^. Importantly, downregulation of DDX3 markedly enhanced the incidence of chromosome misalignment and multipolar mitosis from 10% to 16.1% and 10.8% to 20.3%, respectively, as compared to the control (Fig. 2a), which results from dysfunction of supernumerary centrosomes in cancer cells.

In view that mitotic cell containing supernumerary centrosomes or other defects delays mitosis^45^, we examined the cell cycle progression by live imaging of HCT116 cells expressing GFP-H2B or mCherry-alpha-tubulin (Supplementary Fig. S2a,b). Knockdown of DDX3 induced multipolar mitosis and prolonged the progression of mitosis to more than 120 minutes from metaphase to separation of daughter chromosomes (Supplementary Fig. S2a) or to cytokinesis (Supplementary Fig. S2b) while the duration of mitosis was less than 60 minutes in the control. To further assess the mitotic delay induced by DDX3 depletion, control and DDX3-knockdown cells were collected every 4 hours for up to 16 hours after release from G1 or S phase synchronization. Only 22% of control cells remained in the G2/M phase while 47% of DDX3-knockdown cells were still trapped at G2/M phase 16 hours after release from G1 arrest (Fig. 2b). Similarly, 21% of control cells remained in the G2/M phase while 34% of DDX3-knockdown cells were still trapped at G2/M phase 16 hours after release from S block (Fig. 2c). Therefore, our results indicate that knockdown of DDX3 delays the G2/M transition. γH2AX is a marker for DNA double strand breaks. Ser^345^-phosphorylated CHK1 and Tyr^15^-phosphorylated CDC2 are negative regulators of G2/M transition and are activated in response to DNA damage^46^. To further confirm the critical roles of DDX3 in mitosis and G2/M transition, the expression of γH2AX, Ser^345^-phosphorylated CHK1 and Tyr^15^-phosphorylated CDC2 in control and DDX3 knockdown cells were examined. Knockdown of DDX3 increased γH2AX expression to 1.48-fold, indicating that knockdown of DDX3 increases DNA damage. Moreover, knockdown of DDX3 induced a 3.83-fold activation of phosphorylation of CHK1 at Ser^345^ and also caused a 2.4-fold accumulation of Tyr^15^-phosphorylated CDC2 (Fig. 2d), which confirms that depletion of DDX3 results in G2/M phase arrest.

Since severe DNA damage elicits cell death to eliminate damaged cell^47^, we investigated the sub-G1 population of the control and DDX3-knockdown cells. Downregulation of DDX3 resulted in a marked increase in the sub-G1 peak from 10% to 19% as compared to the control (Fig. 2e). Moreover, knockdown of DDX3 reduced cell proliferation rate as compared to the control (Supplementary Fig. S2d). Taken together, these results suggest that knockdown of DDX3 causes mitotic defects and DNA damage, leading to G2/M delay and cell death.

### Downregulation of DDX3 leads to multipolar mitosis by impairing centrosome inactivation and clustering

**Figure 3:**
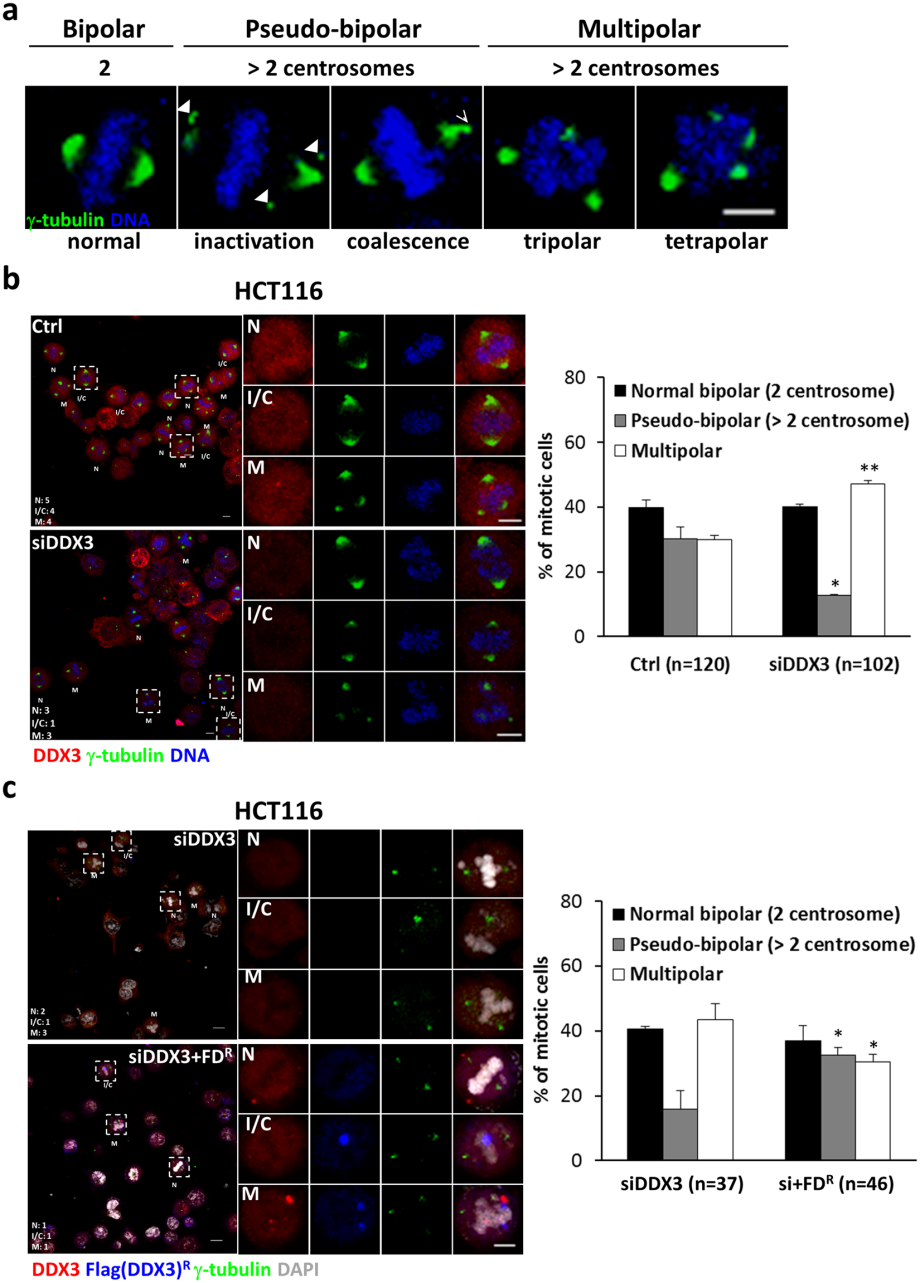
DDX3 specifically prevents multipolar mitosis by clustering or inactivating extra centrosomes. (**a**) Representative confocal images showing bipolar and multipolar mitosis. Cells with more than two centrosomes can undergo multipolar mitosis or bipolar mitosis through inactivation (arrowheads) or coalescence (arrow) of the extra centrosomes. Cells were immunostained with γ-tubulin (green) and DNA (blue). Images are shown as the maximum projections of confocal Z stacks. Scale bar = 5 μm. (**b**) Depletion of DDX3 impaired the centrosome inactivation/coalescence and promoted multipolar mitosis in HCT116 cells. Confocal images of control or DDX3-knockdown mitotic cells classified as normal bipolar mitosis (N), pseudo-bipolar mitosis by centrosome inactivation/clustering (I/C) or multipolar mitosis (M). Each typical mitotic cell is shown at higher magnification in the right column. Cells were immunostained with DDX3 (red), γ-tubulin (green) and DNA (blue). Images are shown as the maximum projections of confocal Z stacks. Scale bar = 5 μm. The proportions of bipolar mitosis (two centrosomes), pseudo-bipolar mitosis (more than two centrosomes) and multipolar mitosis in the control and DDX3-knockdown cells were statistically analyzed (right panel). Data are shown as average value ± S.D. calculated from two independent experiments. *P < 0.05; **P < 0.01. (n), the number of cells analyzed. (**c**) Reintroduction of shDDX3-resistant Flag-DDX3 rescued the centrosome inactivation/coalescence and prevented multipolar mitosis in the DDX3-knockdown HCT116 cells. Cells were immunostained with DDX3 (red), Flag (blue), γ-tubulin (green) and DNA (gray). Images are shown as the maximum projections of confocal Z stacks. Scale bar = 5 μm. The proportions of mitosis in the DDX3-knockdown and DDX3 overexpressed DDX3-knockdown (si+FD^R^) cells were statistically analyzed (right panel). Data are shown as average value ± S.D. calculated from two independent experiments. *P < 0.05. (n), the number of cells analyzed.

**Figure 4:**
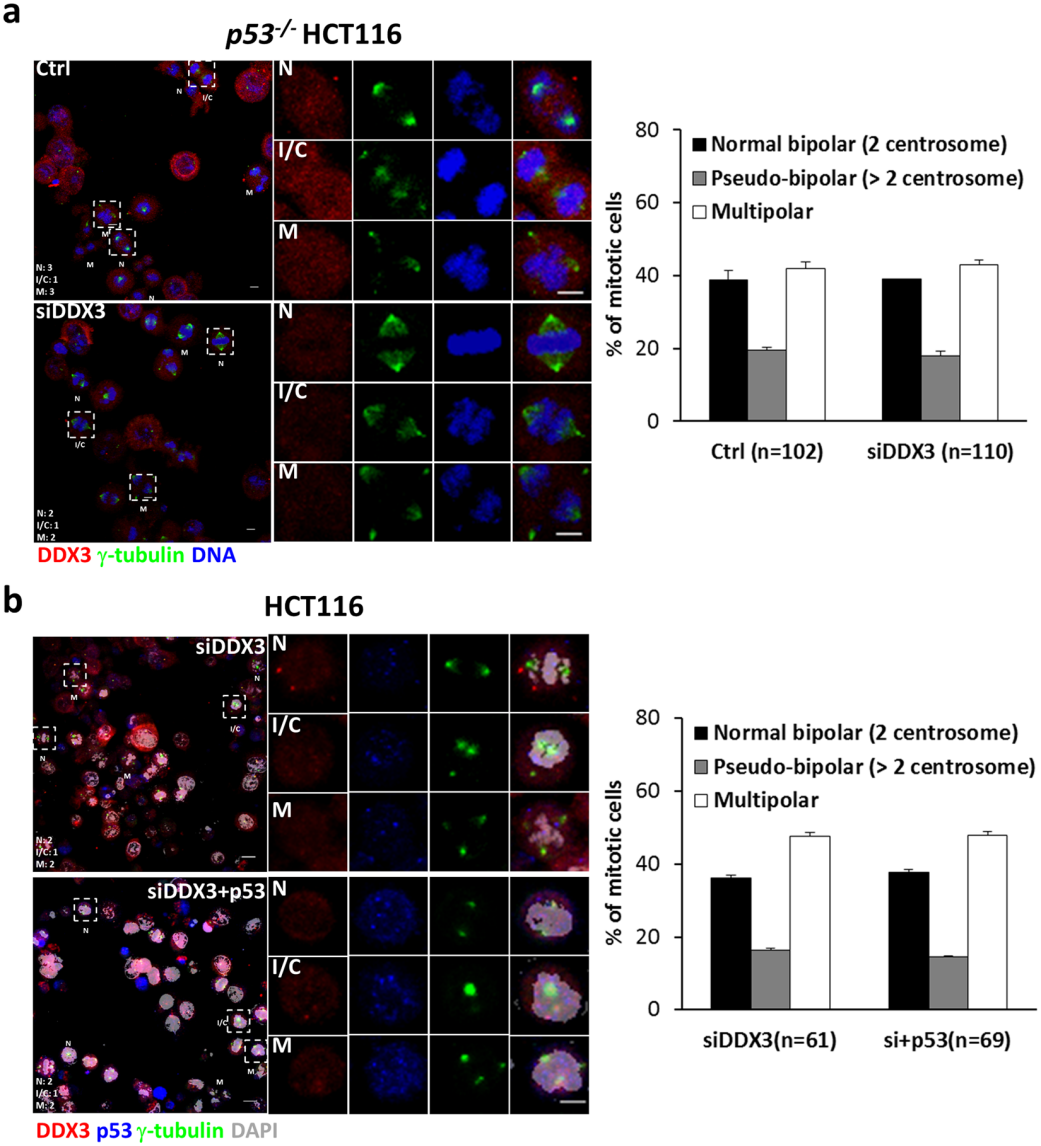
Depletion of DDX3 in *p53* ^−/−^ HCT116 cells and reintroduction of p53 in the DDX3-knockdown HCT116 cells elicited no effect on the multipolar mitosis. (**a**) Depletion of DDX3 elicited no effect on the multipolar mitosis in *p53* ^−/−^ HCT116 cells. Confocal images of control or DDX3-knockdown mitotic cells classified as normal bipolar mitosis (N), pseudo-bipolar mitosis by centrosome inactivation/clustering (I/C) or multipolar mitosis (M). Each typical mitotic cell is shown at higher magnification in the right column. Cells were immunostained with DDX3 (red), γ-tubulin (green) and DNA (blue). Images are shown as the maximum projections of confocal Z stacks. Scale bar = 5 μm. The proportions of bipolar mitosis (two centrosomes), pseudo-bipolar mitosis (more than two centrosomes) and multipolar mitosis in the control and DDX3-knockdown cells were statistically analyzed (right panel). Data are shown as average value ± S.D. calculated from two independent experiments. (n), the number of cells analyzed. (**b**) Reintroduction of p53 was insufficient to rescue the multipolar mitosis caused by knockdown of DDX3 in HCT116 cells. Cells were immunostained with DDX3 (red), p53 (blue), γ-tubulin (green) and DNA (gray). Images are shown as the maximum projections of confocal Z stacks. Scale bar = 5 μm. Data are shown as average value ± S.D. calculated from two independent experiments. (n), the number of cells analyzed.

To get insights into how depletion of DDX3 promoted multipolar mitosis, mitotic cells were characterized as normal bipolar mitosis with two centrosomes, pseudo-bipolar mitosis by inactivation or coalescence of excess centrosomes, or multipolar mitosis by immunostaining with anti-γ-tubulin antibody (Fig. 3a). Knockdown of DDX3 significantly reduced the pseudo-bipolar mitosis (from 30.2% to 12.7%) and increased the multipolar mitosis by 17% (from 29.9% to 47.1%) as compared to the control (Fig. 3b). Reintroduction of DDX3 in DDX3-knockdown cells rescued the percentage of pseudo-bipolar mitosis (from 15.9% to 32.6%) and reduced the multipolar mitosis by 13% (from 43.5% to 30.5%) as compared with knockdown of DDX3 (Fig. 3c). Furthermore, live cell imaging reveals that knockdown of DDX3 increased the proportion of multipolar mitosis from 26% to 59% and also enhanced cell death from 9% to 35%, as compared to the control (Supplementary Fig. S2c). Reintroduction of DDX3 in DDX3-knockdown cells reduced the incidence of multipolar mitosis and cell death to 22% and 8%, respectively. These results demonstrate that DDX3 specifically suppresses multipolar mitosis by inactivation or coalescence of excess centrosomes and prevents cell death. Since DDX3 associates with p53 at centrosome (Fig. 1d,e), and p53 has been reported to be essential for centrosome clustering and preventing multipolar mitosis^11–13^, we examined whether DDX3 prevents multipolar mitosis via p53. DDX3 was depleted in *p53* ^−/−^ HCT116 cells and the mitotic cells were analyzed by immunofluorescence with anti-γ-tubulin antibody. Similar proportions of mitotic cells in the control and DDX3-knockdown *p53* ^−/−^ HCT116 cells were detected (38.7–39.1% with bipolar mitosis, 19.5–18.0% with pseudo-bipolar mitosis and 41.8–43.0% with multipolar mitosis) (Fig. 4a). However, reintroduction of p53 elicited no effect on the multipolar mitosis in the DDX3-knockdown HCT116 cells (36.1–37.7% with bipolar mitosis, 16.4–14.5% with pseudo-bipolar mitosis and 47.5–47.8% with multipolar mitosis) (Fig. 4b), demonstrating that reintroduction of p53 is insufficient to rescue the multipolar mitosis caused by depletion of DDX3.

### Centrosomal localization of DDX3 requires p53 but is independent of the centrosomal localization of p53

**Figure 5:**
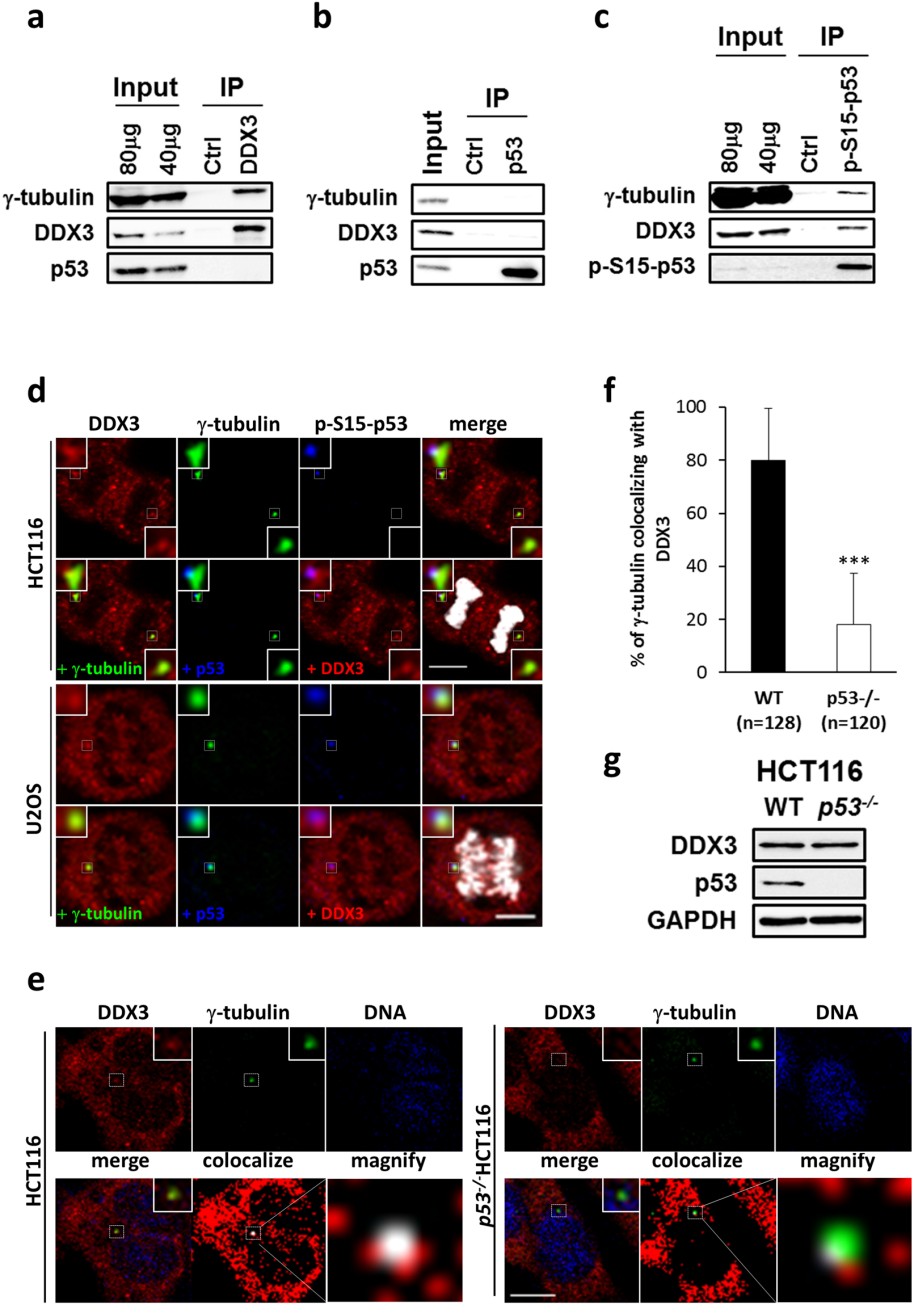
p53 is essential for DDX3 localization at centrosome. (**a**) Western blot analysis showing co-immunoprecipitation of γ-tubulin with DDX3 in HCT116 cells. Original images of western blots were presented in Supplementary Fig. S4a. (**b**) Western blot analysis showing that neither γ-tubulin nor DDX3 co-immunoprecipitates with p53 in HCT116 cells. Original images of western blots were presented in Supplementary Fig. S4b. (**c**) Western blot analysis showing co-immunoprecipitations of γ-tubulin and DDX3 with Ser^15^-phosphorylated p53 in HCT116 cells. Original images of western blots were presented in Supplementary Fig. S4c. (**d**) Representative confocal images show colocalization of DDX3 with Ser^15^-phosphorylated p53 in the centrosome at anaphase. HCT116 and U2OS cells were immunostained with anti-DDX3 (red), anti-γ-tubulin (green), anti-Ser^15^-phosphorylated p53 (blue) antibodies and DAPI (gray). Insets show higher magnifications of the centrosome. Scale bar = 5 μm. (**e**) Representative confocal images showing the level of colocalization between DDX3 and γ-tubulin is significantly reduced in the absence of p53. WT and *p53* ^−/−^ HCT116 cells were immunostained with DDX3 (red), γ-tubulin (green) and DNA (blue). Insets and dashed outline show higher magnification of the centrosome. The colocalized area is displayed in white. Scale bar = 10 μm. (**f**) The percentage of γ-tubulin colocalized with DDX3 in *p53* ^−/−^ HCT116 cells is significantly reduced when compared with WT HCT116 cells. The colocalization of DDX3 and γ-tubulin was analyzed using Zeiss ZEN 2009 software and shown as average value ± S.D. calculated from three independent experiments. The value of 100 indicates perfect co-localization. ***P < 0.001. (n), the number of cells analyzed. (**g**) Western blot analysis showing no difference in DDX3 level between WT and *p53* ^−/−^ HCT116 cells. Original images of western blots were presented in Supplementary Fig. S4d.

**Figure 6:**
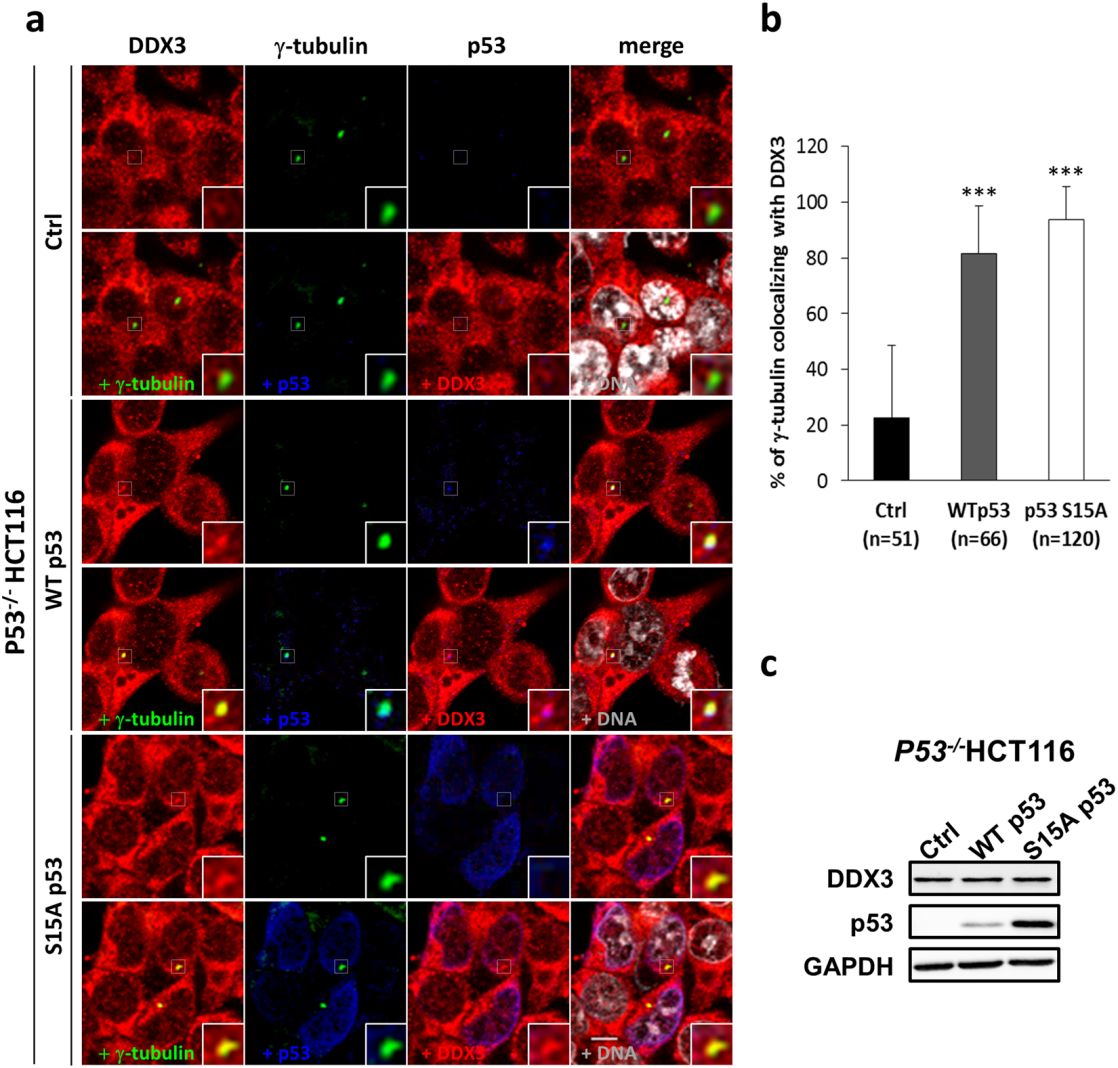
Overexpression of p53 rescues the centrosomal localization of DDX3 in *p53* ^−/−^ HCT116 cells. (**a**). Representative confocal images demonstrate that centrosomal localization of DDX3 is restored after introduction of p53 wild-type (WT) or p53 S15A mutant (S15A) in *p53* ^−/−^ HCT116 cells. The control, p53 WT- or p53 S15A mutant-overexpressed *p53* ^−/−^ HCT116 cells were immunostained with anti-DDX3 (red), anti-γ-tubulin (green), anti-p53 (blue) antibodies and DNA (gray). Insets exhibit higher magnification of the centrosome. Scale bar = 5 μm. (**b**) Overexpression of p53 WT and p53 S15A mutant significantly enhances the percentage of γ-tubulin colocalizing with DDX3 in *p53* ^−/−^ HCT116 cells. The colocalization of DDX3 and γ-tubulin was analyzed using Zeiss ZEN 2009 software and shown as means ± S.D. from three independent experiments. The value of 100 indicates perfect co-localization. ***P < 0.001. (n), the number of cells analyzed. (**c**) Western blot analysis showing the level of DDX3 and p53 in the control, p53 WT- and p53 S15A mutant-overexpressed *p53* ^−/−^ HCT116 cells. GAPDH was used as an internal control. Original images of western blots were presented in Supplementary Fig. S5a.

Since DDX3 localizes to centrosome throughout the cell cycle and associates with p53 at centrosome (Fig. 1), we examined the interaction between DDX3, γ-tubulin and p53 by co-immunoprecipitation assay in HCT116 cell. γ-tubulin was co-immunoprecipitated with DDX3 *in vivo* (Fig. 5a). Coincident with the essential role of serine 15 phosphorylation of p53^7^, DDX3 and γ-tubulin were specifically co-immunoprecipitated with Ser^15^-phosphorylated p53 but not total p53 *in vivo* (Fig. 5b,c). Immunofluorescence assay revealed that DDX3 was also colocalized with Ser^15^-phosphorylated p53 at centrosome during anaphase in HCT116 and U2OS cells (Fig. 5d). To examine the impact of p53 on DDX3 recruitment to centrosome, we estimated the degree of colocalization between DDX3 and γ-tubulin in parental and *p53* ^−/−^ HCT116 cells. Immunofluorescence staining of γ-tubulin was almost fully overlapped with that of DDX3 in parental HCT116 cells but only a limited overlap was detected in *p53* ^−/−^ HCT116 cells (Fig. 5e). Quantitative analysis showed that the percentage of γ-tubulin colocalized with DDX3 was significantly reduced from 80% to 18% in *p53* ^−/−^ HCT116 cells as compared to parental cells (Fig. 5f) while similar levels of expression of DDX3 in parental and *p53* ^−/−^ HCT116 cells were detected (Fig. 5g), suggesting that p53 is essential for DDX3 localization to centrosome. To further examine whether DDX3 is recruited to centrosome by p53, immunofluorescence analysis was performed on *p53* ^−/−^ HCT116 cells expressing p53 wild-type (WT) or centrosomal targeting-defective p53 mutant (S15A). p53 WT was colocalized with DDX3 and γ-tubulin while p53 S15A mutant concentrated in the nucleus without entering the centrosome (Fig. 6a). Quantitative analysis showed that overexpression of p53 WT or p53 S15A mutant significantly enhanced the percentage of γ-tubulin colocalizing with DDX3 from 22% to 81% or 94%, respectively, as compared to the control (Fig. 6b) while the overexpression level of p53 S15A mutant was higher than that of p53 WT in the *p53* ^−/−^ HCT116 cells (Fig. 6c). Our results demonstrate that centrosomal localization of DDX3 requires p53 but not through association with p53 at centrosome.

### Depletion of DDX3 suppresses the level of Ser^15^-phosphorylated p53 and its centrosomal localization

**Figure 7:**
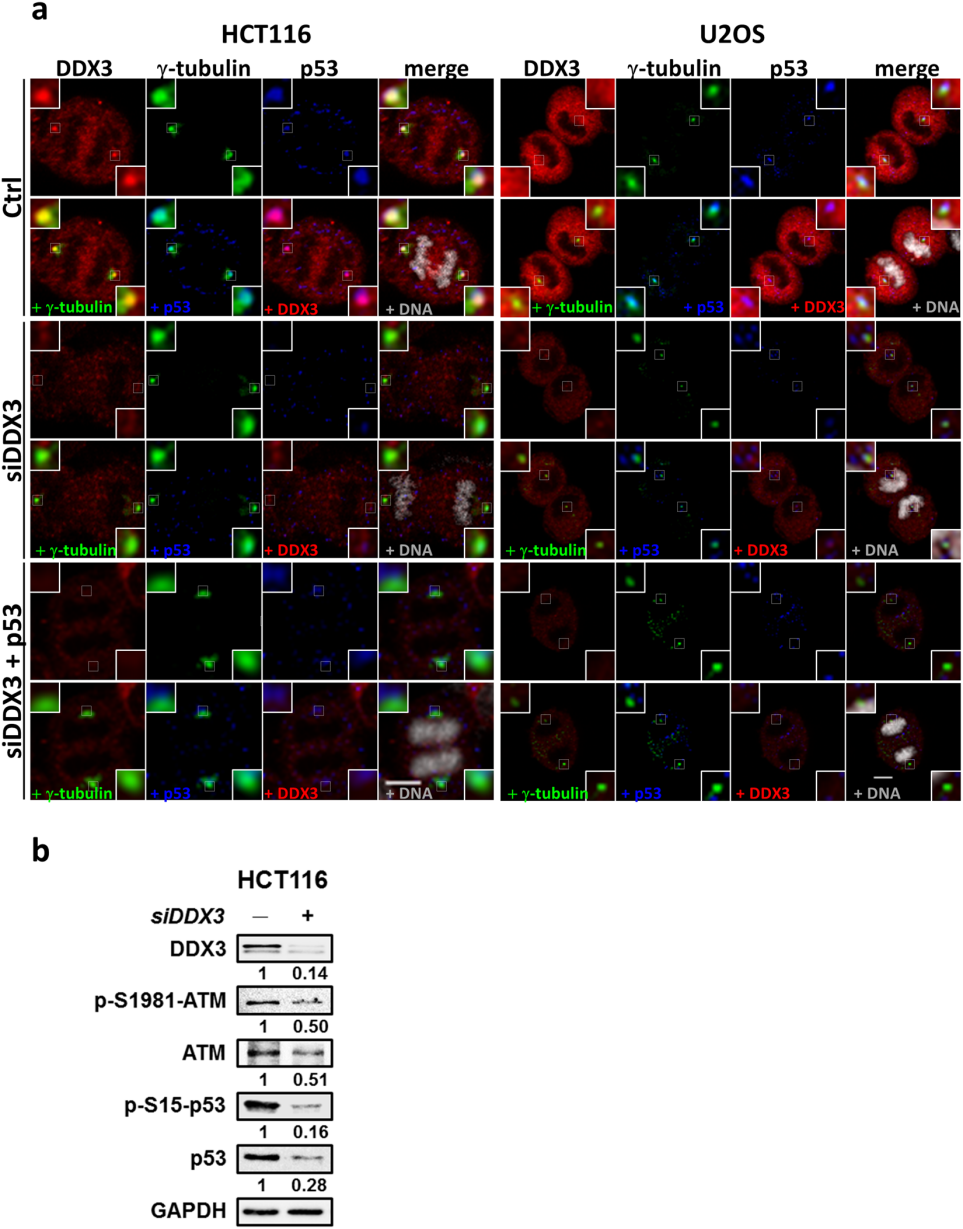
Knockdown of DDX3 results in displacement of p53 from centrosome. (**a**) Representative confocal images demonstrate that DDX3 is required for p53 localization at centrosome in both HCT116 and U2OS cells. The control, DDX3-knockdown and p53-overexpressed DDX3-knockdown cells were immunostained with anti-DDX3 (red), anti-γ-tubulin (green), anti-p53 (blue) antibodies and DNA (gray). Insets exhibited higher magnification of the centrosome. Scale bar = 5 μm. (**b**) Western blot analysis showing DDX3 knockdown suppressed the expression of ATM, phosphor-Ser^1981^-ATM, phosphor-Ser^15^-p53 and p53. Original images of western blots were presented in Supplementary Fig. S5b.

To elucidate the impact of DDX3 on p53 recruitment to centrosome, we examined colocalization between p53 and γ-tubulin in the control and DDX3-knockdown HCT116 and U2OS cells. Immunofluorescence staining of p53 was fully overlapped with that of γ-tubulin during anaphase in the control cells whereas knockdown of DDX3 reduced the colocalization between p53 and γ-tubulin (Fig. 7a). Moreover, reintroduction of p53 in the DDX3-knockdown cells could not rescue the colocalization between p53 and γ-tubulin, indicating that DDX3 is essential for p53 centrosomal localization. Since phosphorylation of p53 at Serine 15 is required for centrosomal targeting of p53, we further examined whether depletion of DDX3 affects the level of Ser^15^-phosphorylated p53 and ATM kinase, which phosphorylates p53 at Ser^15^. Knockdown of DDX3 suppressed the expression of Ser^1981^-phosphorylated ATM by 0.5-fold. Depletion of DDX3 also suppressed Ser^15^ phosphorylation of p53 to 0.16-fold while the total p53 was reduced to 0.28-fold (Fig. 7b). Therefore, our results demonstrate that depletion of DDX3 suppresses the centrosomal localization of p53 through inhibition of p53 expression, particularly the phosphorylation of p53 at Ser^15^ because DDX3 knockdown also induced the inhibition of ATM kinase.

### Knockdown of DDX3 suppresses p53 expression by inhibiting p53 mRNA translation but not p53 stability

**Figure 8:**
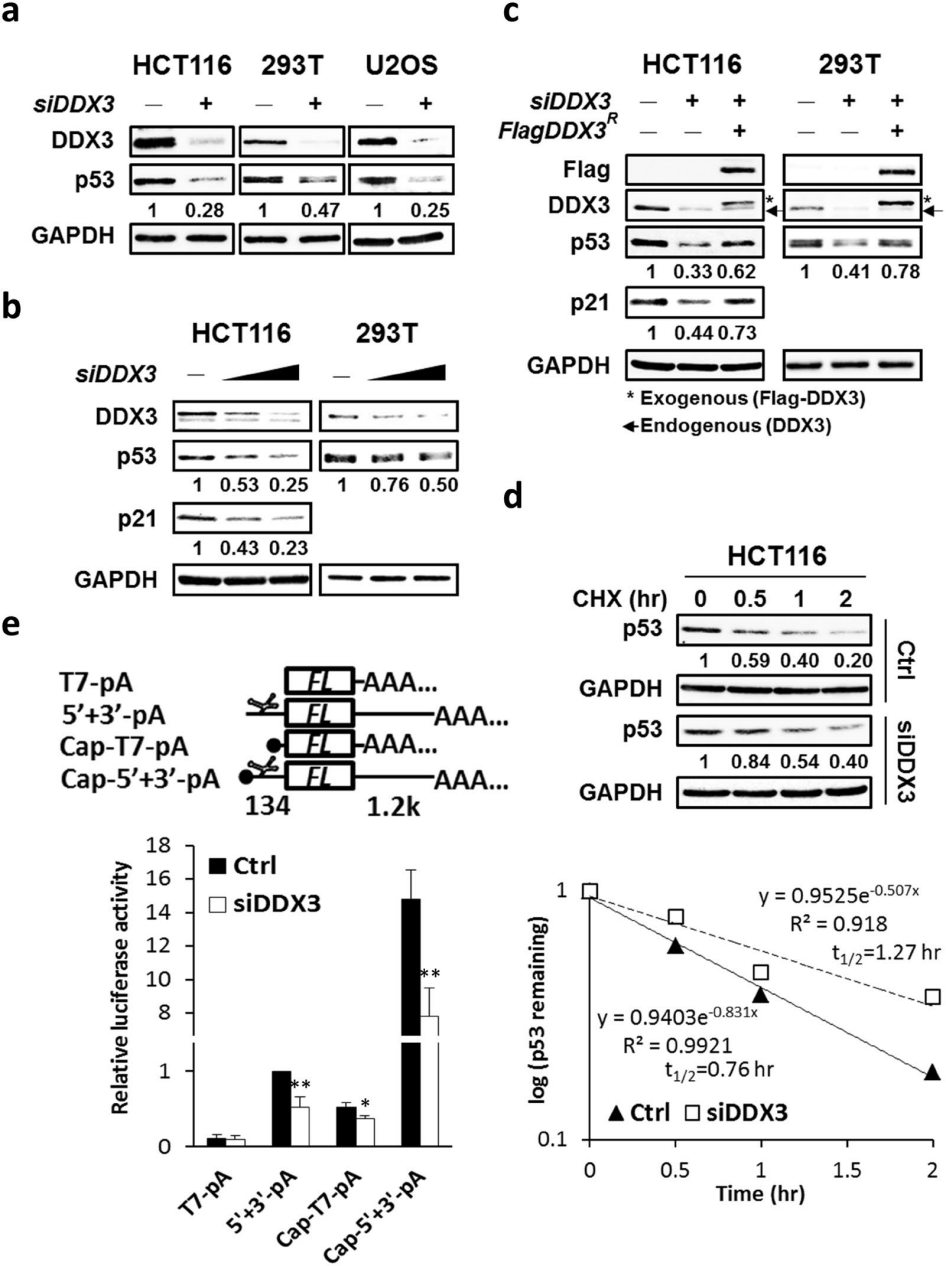
Knockdown of DDX3 suppresses p53 expression by inhibiting *p53* mRNA translation but not p53 stability. (**a**) Western blot analysis reveals DDX3 knockdown repressed p53 expression in HCT116, 293 T and U2OS cells. Original images of western blots were presented in Supplementary Fig. S6a. (**b**) Western blot analysis reveals dose-dependent knockdown of DDX3 resulted in differential repression of p53 or p21 in HCT116 and 293 T cells. Original images of western blots were presented in Supplementary Fig. S6b. (**c**) Western blot analysis reveals ectopic expression of shDDX3-resistant Flag-DDX3 partially rescued p53 or p21 in the DDX3-knockdown HCT116 and 293 T cells. Original images of western blots were presented in Supplementary Fig. S6c. (**d**) Western blot analysis reveals DDX3 depletion resulted in reduced p53 degradation. Control and DDX3-knockdown HCT116 cells were incubated with 40 μg/ml cycloheximide for indicated time points and the half-life of p53 was analyzed. Original images of western blots were presented in Supplementary Fig. S7a. (**e**) Schematic diagrams of luciferase reporter RNAs. *T7-pA* and *5*′ + *3*′*-pA* are uncapped reporter RNAs whereas *Cap-T7-pA* and *Cap-5*′ + *3*′*-pA* are capped reporter RNAs. In the *5*′ + *3*′*-pA* and *Cap-5*′ + *3*′*-pA* reporter RNAs, the *p53 IRES* (134 bp) is inserted upstream and *p53 3*′*UTR* (1.2k bp) is inserted downstream of the *firefly* luciferase gene (*FL*). Knockdown of DDX3 suppressed the *p53* IRES-mediated translation and cap-dependent translation of reporter RNAs. Data are shown as average value ± S.D. calculated from three independent experiments performed in triplicate. **P < 0.01; *P < 0.05.

To confirm the positive regulatory role of DDX3 in p53 expression, p53 protein levels were analyzed in DDX3-knockdown HCT116 colorectal cancer cells, human embryonic kidney 293T cells and osteosarcoma U2OS cells. Depletion of DDX3 suppressed p53 expression to 0.25–0.47-fold in HCT116, 293T and U2OS cells (Fig. 8a). Additionally, knockdown of DDX3 suppressed p53 expression in a dose-dependent manner in HCT116 and 293T cells, and also dose-dependently suppressed the expression of the p53 target gene, p21 in HCT116 cells (Fig. 8b). Ectopic expression of shDDX3-resistant Flag-tagged DDX3 partially rescued p53 expression in DDX3-knockdown HCT116 and 293T cells, as well as the p21 expression in DDX3-knockdown HCT116 cells (Fig. 8c). These data reveal that p53 expression is specifically regulated by DDX3.

To verify whether DDX3 regulates p53 expression by stabilization of p53^37^, we measured the half-life of p53 in control and DDX3-knockdown HCT116 cells by treating cells with the protein synthesis inhibitor cycloheximide. The half-life of p53 was extended from 0.76 to 1.27 hour by knockdown of DDX3 in HCT116 cells (Fig. 8d), suggesting that the upregulation of p53 expression by DDX3 is not through stabilization of p53 in HCT116 cells. In view that DDX3 is involved in cellular mRNA translation and viral IRES-dependent translation^48–50^, we next examined whether DDX3 regulates translation of *p53* mRNA by RNA reporter assay. To mimic cellular *p53* mRNA structure, RNA reporter plasmids were constructed by inserting *p53* 5′UTR in the upstream and *p53* 3′UTR in the downstream of the control luciferase reporter gene. Then reporter RNAs, *T7-pA*, *5*′+*3*′*-pA*, *Cap-T7-pA* and *Cap-5*′ + *3*′*-pA* were transcribed *in vitro* (Fig. 8e). Knockdown of DDX3 suppressed the *Cap-T7-pA* reporter activity to 0.72-fold while having no effect on the control *T7-pA*, indicating that DDX3 modulates general cap-dependent translation, which is consistent with previous studies^29, 49^. Knockdown of DDX3 further suppressed the *Cap-5*′ + *3*′*-pA* reporter activity to 0.53-fold, demonstrating that DDX3 specifically regulates *p53* mRNA translation. Furthermore, depletion of DDX3 suppressed *5*′ + *3*′*-pA* reporter activity to 0.53-fold while it had no effect on the control *T7-pA*, implying that DDX3 modulates *p53* IRES-mediated translation. Altogether, these results reveal that DDX3 positively regulates p53 expression through activating *p53* mRNA translation but not p53 stability.

### Downregulation of DDX3 inhibits p53 transcription through activation of DNMTs and hypermethylation of p53 promoter

**Figure 9:**
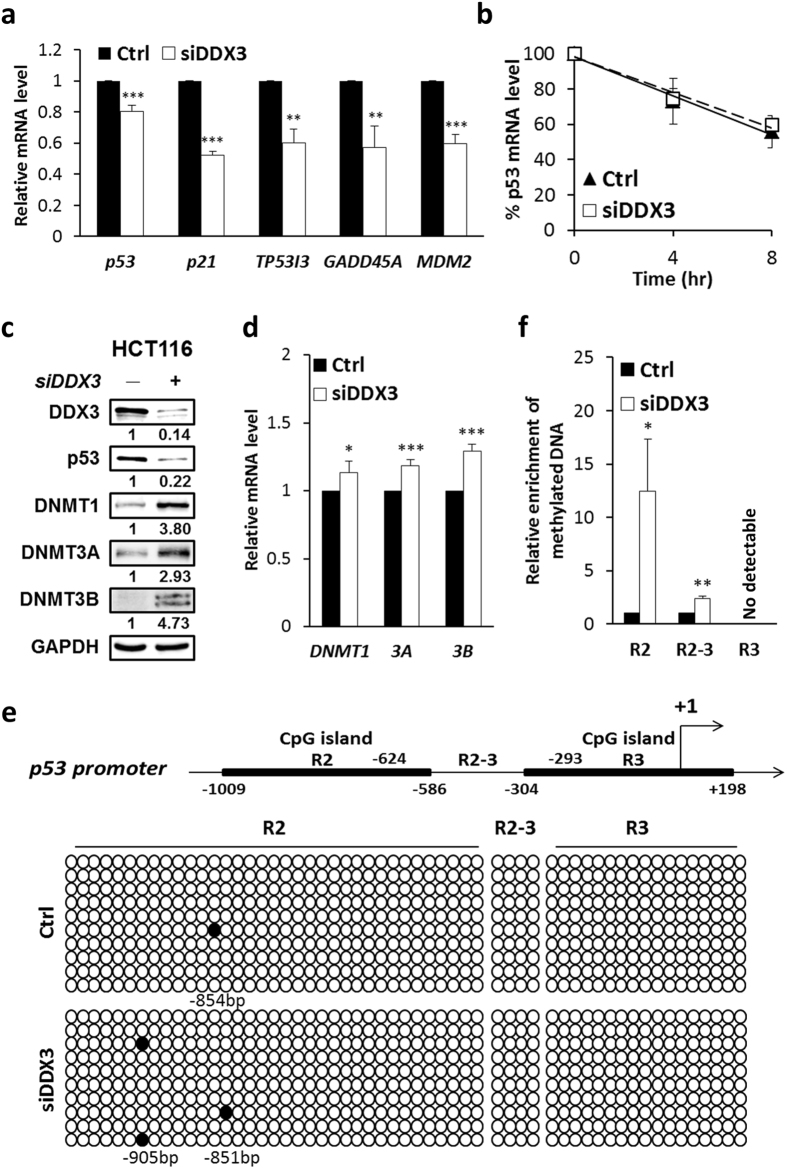
Knockdown of DDX3 inhibits *p53* transcription through activation of the DNMTs and DNA hypermethylation. (**a**) Depletion of DDX3 suppressed the mRNA expression of *p53* and target genes, *p21*, *TP53I3*, *GADD45A* and *MDM2* in HCT116 cells. Quantitative real-time PCR analysis of *p53* and *p53* target genes were normalized to *GAPDH* and shown as average value ± S.D. calculated from three independent experiments. ***P < 0.001; **P < 0.01. (**b**) Analysis of the *p53* mRNA stability in control and DDX3-knockdown HCT116 cells by incubation with 10 μg/ml actinomycin D at indicated time periods. Data were normalized to *GAPDH* and shown as average value ± S.D. calculated from at least two independent experiments. (**c**) Western blot analysis reveals DDX3 knockdown promoted the expression of DNMT1, DNMT3A and DNMT3B in HCT116 cells. Original images of western blots were presented in Supplementary Fig. S7b. (**d**) Quantitative real-time PCR analysis reveals DDX3 depletion increased the mRNA expression of *DNMT1, DNMT3A* and *DNMT3B* in HCT116 cells. Data were normalized to *GAPDH* and shown as average value ± S.D. calculated from three independent experiments. ***P < 0.001; *P < 0.05. (**e**) Knockdown of DDX3 induced *p53* promoter (R2) hypermethylation detected by bisulfide sequencing PCR analysis. Schematic representation of *p53* promoter harboring two CpG islands R2 (from -1009 to −586 bp) and R3 (from −304 to + 198 bp) relative to transcription start site (TSS) (+1). Open circles denote unmethylated CG sites, filled circles are methylated CG sites. Ten independent clones were sequenced in each case. (**f**) DDX3 deficiency induced hypermethylation in *p53* promoter (R2) detected using EpiMark methylated DNA enrichment kit. Data are shown as average value ± S.D. calculated from at least two independent experiments. **P < 0.01; *P < 0.05.

To explore whether DDX3 regulates *p53* transcription, we detected the mRNA expression level of *p53* by quantitative real-time RT-PCR. Depletion of DDX3 suppressed the mRNA expression of *p53* (0.8-fold) as well as p53 downstream target gene *p21*, *TP53I3*, *GADD45A* and *MDM2* (0.52–0.6-fold) (Fig. 9a). Moreover, to examine whether DDX3 modulates *p53* mRNA levels by regulating *p53* mRNA stability, *p53* mRNA level was assessed in control and DDX3-knockdown cells treated with transcription inhibitor, actinomycin D for 4 or 8 hours. Our results show that depletion of DDX3 did not affect *p53* mRNA stability (Fig. 9b), indicating that reduction of DDX3 suppresses *p53* transcription.

Given that *p53* transcription is mediated by epigenetic control^17^ and that DDX3 has been reported to regulate DNA methyltransferase 3 A expression and promoter methylation in HepG2 cells^32^, we determined the DNA methyltransferases (DNMTs) expression and methylation status of *p53* promoter in DDX3-knockdown HCT116 cells. Depletion of DDX3 stimulated the expression of DNMT1, DNMT3A and DNMT3B by 3.80-, 2.93- and 4.73-fold compared with that of control cells, respectively (Fig. 9c). Additionally, knockdown of DDX3 slightly enhanced the mRNA expression of *DNMT1, DNMT3A* and *DNMT3B* by 1.1 to 1.3-fold as compared with the control (Fig. 9d), indicating that DDX3 negatively regulates protein and mRNA expression of DNMTs in HCT116 cells. Furthermore, *p53* promoter contains two CpG islands, R2 (−1009 to −586 bp) and R3 (−304 to + 198 bp) (Fig. 9e). Knockdown of DDX3 resulted in hypermethylation of p53 promoter region R2 but no DNA methylation was detected in region R2–3 and R3 by bisulfite sequencing PCR (Fig. 6e). Consistently, by using EpiMark methylated DNA enrichment kit, an approximately 12-fold increase in DNA methylation in region R2 and a 2.4-fold increase in region R2–3 were detected in DDX3-knockdown HCT116 cells while no methylated DNA was detected in region R3 (Fig. 9f). These results demonstrate that knockdown of DDX3 suppresses *p53* transcription by increasing DNMTs expression and inducing *p53* promoter hypermethylation.

### Depletion of DDX3 enriches the binding of DNMTs and repressive histone marks to p53 promoter

**Figure 10:**
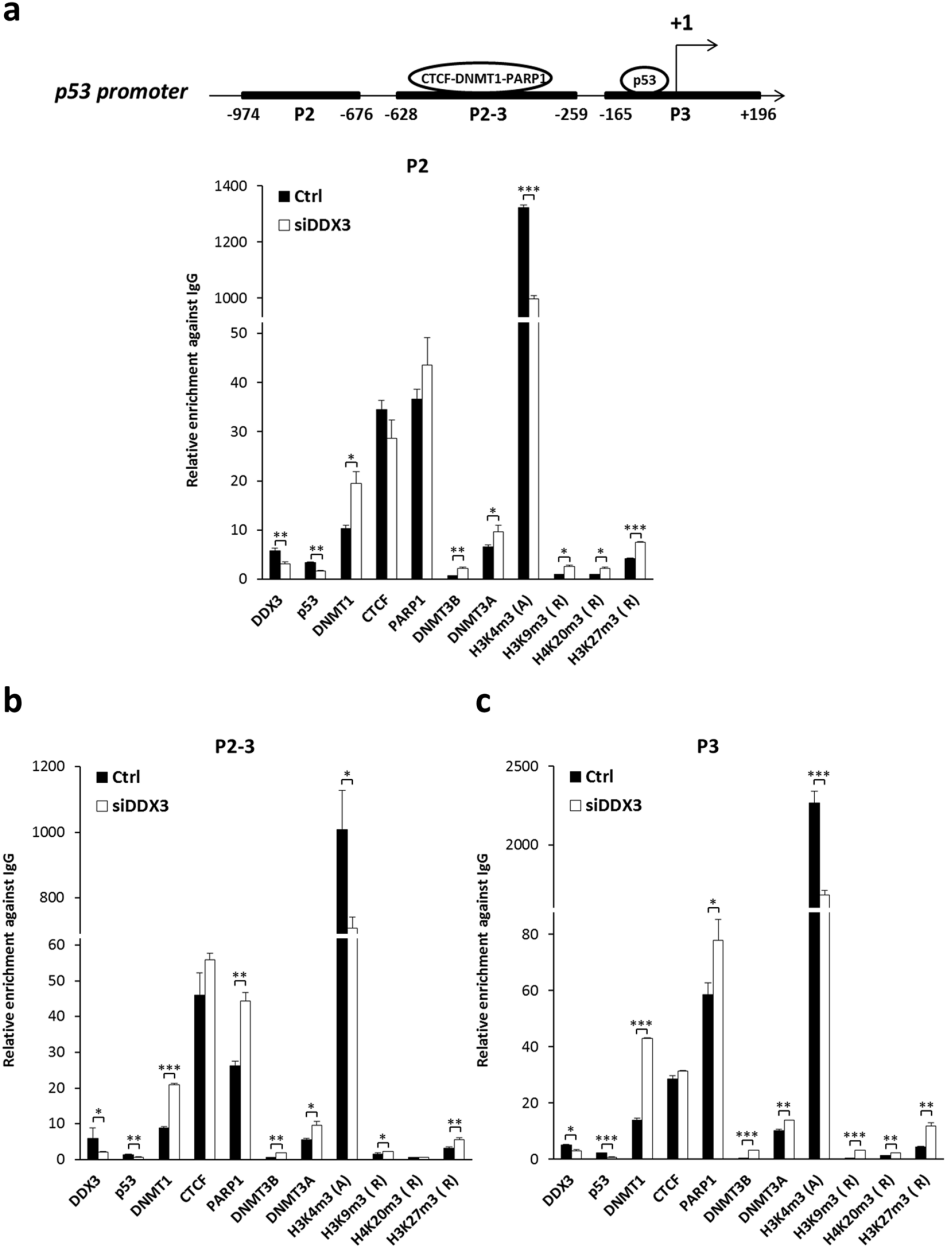
Depletion of DDX3 enriches the binding of DNMTs and repressive histone marks to *p53* promoter. (**a**–**c**) Schematic representation of *p53* promoter containing CTCF-DNMT1-PARP1 complex binding sites in region p2-3 (from −628 to −259 bp)^18^ and p53 binding site in region p3 (from −165 to + 196 bp)^15^, respectively. DDX3 knockdown enhanced the binding abilities of DNMT1, DNMT3A, DNMT3B and repressive histone marks on *p53* promoter and reduced the binding of p53 and active H3K4me3 histone mark. For chromatin immunoprecipitation assay, immunoprecipitates were analyzed by quantitative real-time PCR with specific primers for region p2 (**a**) p2-3 (**b**) and p3 (**c**) on *p53* promoter. The relative binding activity of each protein on *p53* promoter was normalized with input DNA and present as relative fold change against control rabbit IgG. Data are shown as average value ± S.D. calculated from at least two independent experiments. ***P < 0.001; **P < 0.01; *P < 0.05.

To investigate which DNA methyltransferase is responsible for *p53* promoter methylation, the binding activity of DNMTs, CTCF and PARP1 were analyzed by chromatin immunoprecipitation assay (ChIP) in DDX3-knockdown HCT116 cells. Depletion of DDX3 significantly enhanced the binding of DNMT1, DNMT3A, and DNMT3B to all three regions of *p53* promoter (Fig. 10a–c). The binding of CTCF was not significantly changed but the binding of PARP1 was elevated in both region p2-3 and p3 in DDX3-knockdown cells (Fig. 10b,c), indicating that PARP1 preserves the methylation-free status in region p2-3 and p3 of *p53* promoter. Furthermore, in view of bidirectional crosstalk between DNA methylation and histone modification^51^, we examined the binding of active and repressive histone marks on *p53* promoter. Depletion of DDX3 reduced active mark H3K4me3 and enhanced repressive marks, H3K9me3, H4K20me3 and H3K27me3 binding to *p53* promoter (Fig. 10a–c). Moreover, knockdown of DDX3 diminished the binding of DDX3 and p53 to *p53* promoter (Fig. 10a–c). Taken together, these findings demonstrate that knockdown of DDX3 suppresses *p53* promoter by enhancing DNMTs and repressive histone marks binding to *p53* promoter.

## Discussion

**Figure 11:**
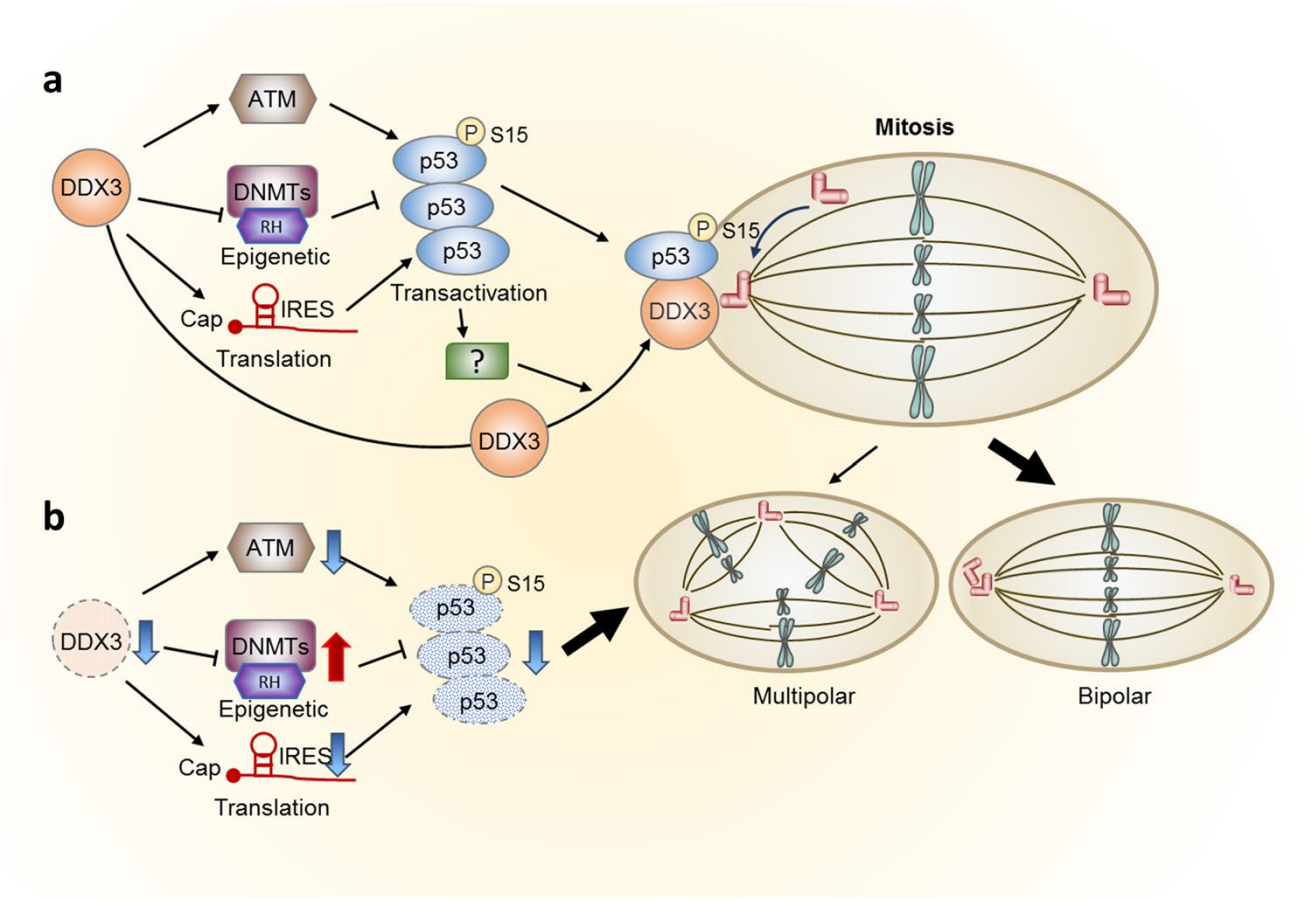
A proposed model illustrates that DDX3 prevents multipolar mitosis through regulation of p53. (**a**) In addition to activation of p53 expression by DDX3 through epigenetic transcriptional and translational regulation, DDX3 promotes phosphorylation of p53 at Ser^15^ by activation of ATM kinase, leading to centrosomal localization of p53. p53 also facilitates the centrosomal localization of DDX3. DDX3 and p53 localizing to centrosome during mitosis promotes centrosome clustering and induces bipolar mitosis to maintain genome stability. (**b**) Knockdown of DDX3 suppresses ATM kinase and phosphorylation of p53 at Ser^15^, resulting in displacement of p53 from the centrosome. Moreover, DDX3 depletion suppresses p53 expression epigenetically by activation of DNMTs and repressive histone marks binding to *p53* promoter, eventually leading to multipolar mitosis and facilitating cell death. (RH, repressive histone marks).

DDX3 plays divergent roles in tumorigenesis^25–27^. Recent studies imply that DDX3 participates in mitosis and loss of DDX3 causes DNA damage and increases cell death^38–40^, but the underlying mechanism remains unclear. In this study, we provided evidence that DDX3 facilitates accurate mitotic progression and prevents mitotic DNA damages (Fig. 2) via localization at centrosome throughout the cell cycle and colocalization with centrosome-associated p53 during mitosis (Fig. 1). P53 is essential for DDX3 recruitment to centrosome (Figs 5 and 6) where DDX3 promotes pseudo-bipolar mitosis by coalescence and inactivation of excess centrosomes to reduce severe aneuploidy (Figs 3–4). DDX3 is also required for centrosomal targeting of p53 through activation of ATM kinase and phosphorylation of p53 at Ser^15^ (Fig. 7). Depletion of DDX3 suppressed *p53* mRNA translation (Fig. 8) and also inhibited *p53* transcription by activation of DNMTs, hypermethylation of p53 promoter (Fig. 9) as well as increased repressive histone marks binding to *p53* promoter (Fig. 10). Therefore, a molecular model for the role of DDX3 in inhibiting multipolar mitosis was proposed (Fig. 11). p53 is required for centrosomal localization of DDX3 which is mediated by transactivation-dependent regulation of p53 but is independent of the centrosomal localization of p53. DDX3 activates p53 expression through epigenetic transcriptional and translational control. Moreover, by activating expression of the ATM kinase, DDX3 promotes phosphorylation of p53 at Ser^15^, which leads to centrosomal localization of p53 with DDX3 at centrosome during mitosis, therefore inducing bipolar mitosis to maintain genome stability. However, DDX3 depletion suppresses p53 expression by activation of DNMTs and repressive histone marks binding to *p53* promoter and also causes displacement of p53 from the centrosome, eventually leading to multipolar mitosis and facilitating cell death.

As noted, the role of DDX3 in colorectal cancer is controversial^27^. Our study provides new insight into a novel role of DDX3 as a tumor suppressor by ensuring proper mitotic progression and maintenance of genome stability through the interplay with tumor suppressor p53 in HCT116 cells. Consistently, DDX3 has been reported to regulate the Snail/E-cadherin pathway to prevent cell migration and invasion in HCT116 cells, which also supports the tumor suppressive role of DDX3^35^. HCT116 is a colorectal cancer cell line that harbors β-catenin mutant but possesses wild-type p53. In contrast, DDX3 has been reported to be positively correlated with β-catenin expression and to promote cell invasion in a subset of colorectal cancer cell lines containing wild-type β-catenin but mutant p53, therefore functioning as an oncogene^33, 34, 52^. Apparently, oncogenic role of DDX3 in colorectal cancer may be attributed to p53 mutations in various cell lines.

Accumulated evidence indicates the bidirectional regulation between DDX3 and p53. For example, p53 activates DDX3 expression in lung cancer cells^36^. Additionally, DDX3 has been reported to interact with p53 and promote p53 accumulation in response to DNA damage in MCF-7 cells^37^. In our study, DDX3 specifically prevents multipolar mitosis by localizing at centrosome and promoting the expression as well as centrosomal localization of p53. p53 is required for centrosomal localization of DDX3, which is mediated by the transactivation regulation of p53 but is independent of the centrosomal localization of p53 (Figs 5–6). Moreover, knockdown of DDX3 elicited no effect on the mitotic defects in the *p53* ^−/−^ HCT116 cells due to loss of centrosomal localization of DDX3 in the *p53* ^−/−^ HCT116 cells (Figs 3–5). Notably, DDX3 depletion suppressed the p53 expression and also suppressed the transactivation regulation of p53 (Figs 8–9a). Furthermore, knockdown of DDX3 inhibited ATM kinase-mediated phosphorylation of p53 at Ser^15^, which results in displacement of p53 from the centrosome (Fig. 7). However, the level of DDX3 in *p53* ^−/−^ HCT116 cells was similar to that in parental HCT116 cells (Fig. 5g). Overexpression of p53 WT and p53 S15A mutant had no effect on DDX3 expression in *p53* ^−/−^ HCT116 cells (Fig. 6c), indicating that DDX3 expression is not modulated by p53 in HCT116 cells. Moreover, DDX3 was co-immunoprecipitated with Ser^15^-phosphorylated p53 but not total p53 in HCT116 cells (Fig. 5b,c). Taken together, the mutually relied centrosomal localization of DDX3 and p53 is independent of the DDX3-p53 interaction.

DDX3 positively regulates the expression of ATM kinase and phosphor-Ser^15^-p53 (Fig. 7b), revealing that DDX3 activates the p53 response to DNA damage, which is consistent with the previous study^37^. Posttranslational modifications on p53 regulate its transactivation, DNA-binding ability and protein stability^55^. For example, phosphorylation of p53 at serine 15 increases interaction with coactivator CBP/p300 which stimulates p53-dependent transactivation of p53 responsive promoters^55^. Consistently, in our study, knockdown of DDX3 inhibited mRNA expression of p53 target genes, *p21, TP53I3, GADD45A* as well as *MDM2* (Fig. 9a) and diminished the binding of p53 to *p53* promoter (Fig. 10). Furthermore, MDM2 is the primary ubiquitin ligase for p53 and promotes proteasomal degradation of p53. Phosphorylation of p53 at serine 15 attenuates MDM2 binding to p53 and thus promotes p53 stability^55^. However, DDX3 has been reported to activate MDM2 expression through interaction with Sp1^36^. These reports indicated that DDX3 positively regulates p53 and MDM2 while p53 and MDM2 form a negative feedback loop. In this study, we observed that knockdown of DDX3 suppressed p53 expression (Fig. 8a–c) and caused a reduction in *MDM2* mRNA level (Fig. 9a) as well as a delay in p53 protein degradation in HCT116 cells (Fig. 8d). This finding implies that knockdown of DDX3 delays p53 degradation likely due to the decreased negative feedback regulation by MDM2. Moreover, we have demonstrated that DDX3 modulates p53 expression through translational (Fig. 8e) and epigenetic (Figs 9–10) regulations but not p53 stability (Fig. 8d) in HCT116 cells.

In this study, we found that DDX3 activates *p53* transcription through modulation of DNMTs (Fig. 9a,c,d). Knockdown of DDX3 enhanced DNMTs expression but only slightly increased the mRNA level of DNMTs (Fig. 9c,d). DNMTs mRNA overexpression is transcriptionally regulated by activation of Sp1 or loss of suppression of p53^56^. DDX3 not only cooperates with Sp1 to activate downstream target genes^31, 36^ but also controls p53 expression (Fig. 8). Consequently, the slightly increased mRNA level of DNMTs (Fig. 9d) in DDX3-knockdown cells may be the result of removal of repression by p53 coupled with low activation of Sp1. Additionally, it has been reported that binding of DNMT3A/3B to methylated DNA helps stabilize DNMT3A/3B^57^. We found that depletion of DDX3 activated DNMTs binding to *p53* promoter (Fig. 10) and thus significantly enhanced DNMTs expression (Fig. 9c). Although knockdown of DDX3 activated DNMTs binding to *p53* promoter, DNA methylation was only detected in region R2 of *p53* promoter (Fig. 9e,f). By chromatin-immunoprecipitation assay, we observed that the binding of PARP1 was activated in region p2-3 and p3 when DDX3 was silenced (Fig. 10b,c). PARP1, an inhibitor of DNMT1, prevents the DNA methyltransferase activity of DNMT1^18^ and thus preserved the methylation-free status in region R2-3 and R3 of *p53* promoter while DNMT1 cooperated with DNMT3A or DNMT3B for de novo methylation in region R2 in DDX3-knockdown cells (Fig. 9e). DNA methylation and histone modification are linked to each other^51^. For example, methylated DNA and methylated DNA binding proteins may recruit histone deacetylase (HDAC) and histone methyltransferase proteins for subsequent histone modification. In addition, histone methyltransferases have been shown to interact with DNMTs and facilitate de novo DNA methylation at target loci^51^. Histone methylation causes temporal and reversible gene silencing while DNA methylation leads to stable gene inactivation^51^. In our study, knockdown of DDX3 promoted DNA methylation of *p53* region R2 (Fig. 9e,f) and enhanced the binding of DNMTs as well as repressive histone marks to region p2 of *p53* promoter (Fig. 10a). Moreover, knockdown of DDX3 suppressed *p53* promoter by increasing the binding of repressive histone marks and DNMTs to *p53* promoter region p2-3 and p3 (Fig. 10b,c) even though DNA methylation was not detectable in region R2-3 and R3 (Fig. 9e,f).

DDX3 has emerged in the last few years, as a new potential therapeutic target for cancer treatment^52–54^. Inhibition of DDX3 in a variety of cancers results in a reduction of tumor cell growth and increases apoptosis. Our study demonstrates that knockdown of DDX3 promotes multipolar mitosis along with severe aneuploidy, leading to cell cycle delay and cell death (Fig. 2), supporting the aforementioned notion. Furthermore, our study reveals a novel role of DDX3 in the maintenance of genome stability through association with tumor suppressor p53 at centrosome during mitosis (Figs 1, 3–7) and modulation of p53 expression (Figs 8–10), which strengthens the tumor suppressive potential of DDX3 and may be helpful for future development of new strategies in cancer therapy.

## Materials and Methods

### Cell culture and transfection

HCT116 and *p53* ^−/−^ HCT116 were cultured in McCoy’s 5A medium supplemented with 10% fetal bovine serum (FBS). HEK293T and U2OS were cultured in Dulbecco’s modified Eagle’s medium (DMEM) supplemented with 10% FBS. Transient transfection of HCT116, *p53* ^−/−^ HCT116 and U2OS cells was performed using lipofectamine 2000 (Invitrogen) according to the manufacturer’s instruction or the calcium phosphate precipitation method in HEK293T.

### Plasmids

For knockdown of DDX3, cells were transfected with psiDDX3-433 derived from pSUPER plasmid as described previously^30^. Plasmid *Flag-DDX3* ^*R*^, which is a siDDX3-resistant Flag-DDX3 expressing construct, was generated as described previously^58^. Plasmid pCMV-p53 WT and pCMV-p53 S15A mutant were gifts from Dr. Sheau-Yann Shieh (Institute of Biomedical Sciences, Academia Sinica, Taiwan). Plasmid pcDNA3-mCherry-α-tubulin was a gift from Dr. Tang K. Tang (Institute of Biomedical Sciences, Academia Sinica, Taiwan). Plasmid GFP-H2B was a gift from Dr. Jun-Yi Chien (Institute of Microbiology and Immunology, National Yang-Ming University, Taipei, Taiwan). Plasmid 5′ + 3′-pA, which directs the expression of reporter RNA, was generated by inserting *HindIII/BamHI*-treated PCR amplified p53 5′UTR (spanning from −134 to −1 related to translation start site)^24^ into *HindIII/BamHI*-treated Luciferase T7 Control DNA vector (Promega Corporation) and then the *SacI*-digested PCR amplified p53 3′UTR (spanning from 1183 to 2369 related to translation start site)^22^ was further ligated with *SacI*-digested p53 5′UTR-Luciferase T7 Control DNA plasmid.

### In vitro RNA synthesis and RNA transfection

Capped reporter RNAs were transcribed *in vitro* using the mMESSAGE mMACHINE T7 Kit (Ambion) or un-modified reporter RNAs were transcribed using the MEGAscript High-Yield Transcription kit (Ambion) followed by polyadenylation using the poly(A) Tailing kit (Ambion). Synthesized RNAs were further purified using the MEGAclear kit (Ambion). Reporter RNAs were transfected with Lipofectamine 2000 (Invitrogen).

### Antibodies and reagents

The primary antibodies used for immunoprecipitation, immunofluorescence or western blotting were rabbit anti-DDX3^31, 39^, mouse anti-p53 (Calbiochem), mouse anti-p53 (IgG2b) (Santa Cruz Biotech.), rabbit anti-phospho-p53 (Ser^15^) (Cell Signaling Technology), mouse anti-phospho-p53 (Ser^15^) (IgG1) (Cell Signaling Technology), rabbit anti-phospho-ATM (Ser^1981^) (Cell Signaling Technology), rabbit anti-ATM (Cell Signaling Technology), mouse anti-GAPDH (Sigma-Aldrich), mouse anti-p21 (Santa Cruz Biotech.), goat anti-DDDDK (Abcam), mouse anti-α-tubulin (Santa Cruz Biotech), mouse anti-γ-tubulin (IgG1) (Sigma-Aldrich), rabbit anti-γ-tubulin Alexa Fluor 488 (Abcam), mouse anti-γH2AX (Ser^139^) (Millipore), rabbit anti-phospho-Chk1 (Ser^345^) (Cell Signaling Technology), mouse anti-CHK1 (Santa Cruz Biotech), rabbit anti-phospho-cdc2 (Tyr^15^) (Cell Signaling Technology), rabbit anti-cdc2 (Santa Cruz Biotech), goat anti-CTCF (Santa Cruz Biotech), goat anti-DNMT1 (Santa Cruz Biotech), rabbit anti-DNMT3A (Santa Cruz Biotech), mouse anti-DNMT3B (Santa Cruz Biotech), mouse anti-PARP1 (Santa Cruz Biotech), rabbit anti-trimethyl-Histone H3 (Lys^4^) (Millipore), rabbit anti-trimethyl-Histone H3 (Lys^9^) (Millipore), rabbit anti-trimethyl-Histone H4 (Lys^20^) (Millipore), rabbit anti-trimethyl Histone H3 (Lys^27^) (Millipore). Secondary antibodies used for immunofluorescence were anti-mouse IgG2b Alexa Fluor 488, anti-rabbit Alexa Fluor 555 (Molecular Probes, Invitrogen) and anti-mouse IgG1 DyLight649 (Jackson ImmunoResearch Laboratories) antibodies. Cycloheximide, actinomycin D, thymidine, nocodazole and L-mimosine were purchased from Sigma-Aldrich; DAPI was purchased from Roche.

### RNA extraction and quantitative real-time RT-PCR

Total RNA was extracted using TRI reagent (Invitrogen), and first-strand cDNA were synthesized using RevertAid First Strand cDNA Synthesis Kit (Fermentas) with an oligo(dT)_18_ primer. Real-time PCR was performed using SYBR® Green PCR Master Mix (Applied Biosystems) and StepOnePlus™ RealTime PCR System (Applied Biosystems). Primers for quantitative real-time RT-PCR were as follows: p53 (F: 5′-GTTCCGAGAGCTGAATGAGG-3′; R: 5′-TCTGAGTCAGGCCCTTCTGT-3′), p21 (F: 5′-TTAGCAGCGGAACAAGGAGTCA-3′; R: 5′-TTACAGGAGCTGGAAGGTGTTTGG-3′), TP53I3 (F:5′-GCTTCAAATGGCAGAAAAGC-3′; R: 5′-AACCCATCGACCATCAAGAG-3′), GADD45A (F: 5′-AACGGTGATGGCATCT GAATGA-3′; R: 5′-TTCCTTCCTGCATGGTTCTTTGT-3′), MDM2 (F: 5′-ATGT CTGTACCTACTGATGGTGCTG-3′; R: 5′-TCAAAAGCAATGGCTTTGGTCT-3′), DNMT1 (F: 5′-TACCTGGACGACCCTGACCTC-3′; R: 5′-CGTGGCATCAAGATGGACA-3′), DNMT3A (F: 5′-TATTGATGAGCGCACAAGAGAGC-3′; R: 5′-GGGTGTTCCAGGGTAACATTGAG-3′), DNMT3B (F: 5′-GGCAAGTTCTCCGAGGTCTCTG-3′; R: 5′-TGGTACATGGCTTTTCGATAGGA-3′), GAPDH (F: 5′-CACCCACTCCTCCACCTTT-3′; R: 5′-TCCACCACCCTGTT GCTGTAG-3′).

### Reporter assay

For RNA reporter assay, cells were transfected with either psiDDX3-433 or parental vector pSuper. At 4 hr post-transfection, cells were washed and incubated for another 48 hr and then transfected with 4 μg purified reporter RNA using Lipofectamine 2000 (Invitrogen). After incubation for 4 hr, cells were harvested for luciferase activity assay.

### Mitotic enrichment and immunofluorescence

The M phase synchronization was performed as previously described^59^. In brief, HCT116 cells were treated with 3 mM thymidine for 24 hr, released in fresh medium for 9 hr and then incubated with 0.3 μM nocodazole for 4 hr. Mitotic cells were washed and collected by mitotic shake-off, re-suspended in complete medium and seeded on sterile glass slides. After incubation for 40 min, cells were fixed with −20 °C-stored methanol/acetone (1:1) and subsequently probed with primary and secondary antibodies. Images were acquired with a Zeiss LSM700 confocal microscope using Plan-Apochromat 63x/1.40 Oil DIC M27 objective. 0.5-μm optical sections in the z-axis were collected. The percentage of colocalization was analyzed using Zeiss Zen 2009 light edition software.

### Co-immunoprecipitation

HCT116 cells were lysed with ice-cold PBS containing 0.5% NP-40 and 1x protease inhibitor cocktail. Cell extracts (500 μg) were pre-incubated with protein G-sepharose (GE Healthcare) for 1 hr at 4 °C. Pre-cleaned cell extracts were then incubated with anti-DDX3 antibody, anti-p53 antibody, anti-phospho-Ser15-p53 antibody or control IgG at 4 °C overnight, followed by addition of 10 μl BSA-blocked protein G-Sepharose beads and incubated for additional 1 hr at 4 °C. Beads were then washed three times with ice-cold PBS, proteins were eluted and subjected to western blotting with anti-γ-tubulin, anti-DDX3, anti-p53 and anti-phospho-Ser15-p53 antibodies.

### Cell cycle synchronization and flow cytometry

For synchronization of HCT116 cells at G1 or S phase, cells were treated with 3 mM thymidine for 17 hr at 4 hr post transfection. Cells were then washed twice with PBS and released into normal fresh medium for 12 hr at 37 °C. Cells were then enriched in G1 phase by incubation with 0.5 mM L-minosine for 15 hr or in S phase by incubation with 3 mM thymidine for additional 17 hr. Cells were then released and collected at the indicated time points. For flow cytometry analysis, cells were treated with trypsin, re-suspended in 1x PBS and fixed with chilled 70% ethanol at − 20 °C overnight. Resuspended cells were stained with 20 μg/ml propidium iodide (Sigma) solubilized in PBS containing 0.1% Triton X-100 and 200 μg/ml DNase-free RNase A for 30 min in dark at room temperature. Stained cells were analyzed using FACSCalibur™ flow cytometer (BD Biosciences) and ModFit LT software.

### Bisulfite sequencing PCR

Genomic DNA was extracted using QIAamp® DNA Mini Kit (Qiagen) and the extracted DNA was bisulfite treated using EZ DNA Methylation-Lightning Kit (Zymo Research). The bisulfite converted *p53* promoter region R2, R2-3 and R3 were amplified with the primer sets: F (R2) 5′-GTGTTTTTTTTTTTTTTTGGGAGTAGGTAGAAG-3′, R (R2) 5′-AACCTAAAAAATAAAATACAAAAAAAATACAAAACCTACT-3′, F (R2-3) 5′-GTAGGTTTTGTATTTTTTTGTATTTTATTTTTTAGG-3′, R (R2-3) 5′-CTCATCAATTAAAATATCATTTTTTAAAAAAACTTTCC-3′,F (R3) 5′-TTAATTGATGAGAAGAAAGGATTTAGTTGAGAG-3′, R (R3) 5′-TCATCAAATTCAATCAAAAACTTACCCAATCCA-3′. The PCR products were purified using illustra™ GFX™ PCR DNA and Gel Band Purification Kit (GE Healthcare). Purified PCR products were used for TA cloning into pT&A^TM^ vector (Yeastern Biotech). Ten independent clones in each case were sequenced.

### Methylated DNA enrichment assay

Genomic DNA was extracted using QIAamp® DNA Mini Kit (Qiagen) and sheared with the Bioruptor® UCD-200 Sonication System. Fragmented DNA was subjected to methylated DNA enrichment reaction using EpiMARK Methylated DNA Enrichment Kit according to manufacturer’s instruction (New England BioLabs). DNA methylation status of the *p53* promoter region R2, R2-3 and R3 were analyzed by qRT-PCR using the primer sets: F (R2) 5′-TCCCGGGAGGAGAGGCGAAC-3′, R (R2) 5′-GCGGGACTCGGTAGGGGGAG-3′, F (R2-3) 5′-CGCAGCAGGTCTTGCACCTC-3′, R (R2-3) 5′-GCTTTTGCGTTTGCTCTCAGC-3′, F (R3) 5′-TTTCCACCCCAAAATGTTAGTA-3′, R (R3) 5′-ATCAAGTTCAGTCAGGAGCTTA-3′.

### Chromatin immunoprecipitation (ChIP) assay

ChIP assay was performed as described previously^60^. The DNA-protein complexes were decrosslinked and DNA fragments were purified using QIAquick PCR purification kit (Qiagen). Purified DNAs were subjected to qRT-PCR. The specific primers for the *p53* promoter region p2, p2-3 and p3 are identical to the primer sets for *p53* promoter region R2, R2-3 and R3 in EpiMARK methylated DNA enrichment assay.

### Statistical analysis

The statistical analysis was conducted with one-tailed Student’s *t* test.

## Electronic supplementary material

Supplementary information
