# Supplementary material for: DDX3 localizes to the centrosome and prevents multipolar mitosis by epigenetically and translationally modulating p53 expression
Source: Sci Rep. 2017 Aug 25;7:9411. doi: 10.1038/s41598-017-09779-w (PMC5573351; doi:10.1038/s41598-017-09779-w)
Supplement: Supplementary file 1 — Supplementary information [file 41598_2017_9779_MOESM1_ESM.pdf]

Scientific Reports
Supplementary information

DDX3 localizes to the centrosome and prevents multipolar mitosis by epigenetically and translationally modulating p53 expression

Wei-Ju Chen, Wei-Ting Wang, Tsung-Yuan Tsai, Hao-Kang Li, Yan-Hwa Wu Lee

Supplementary Figures S1-S7 and Supplementary Methods

Supplementary Figure and Figure legend

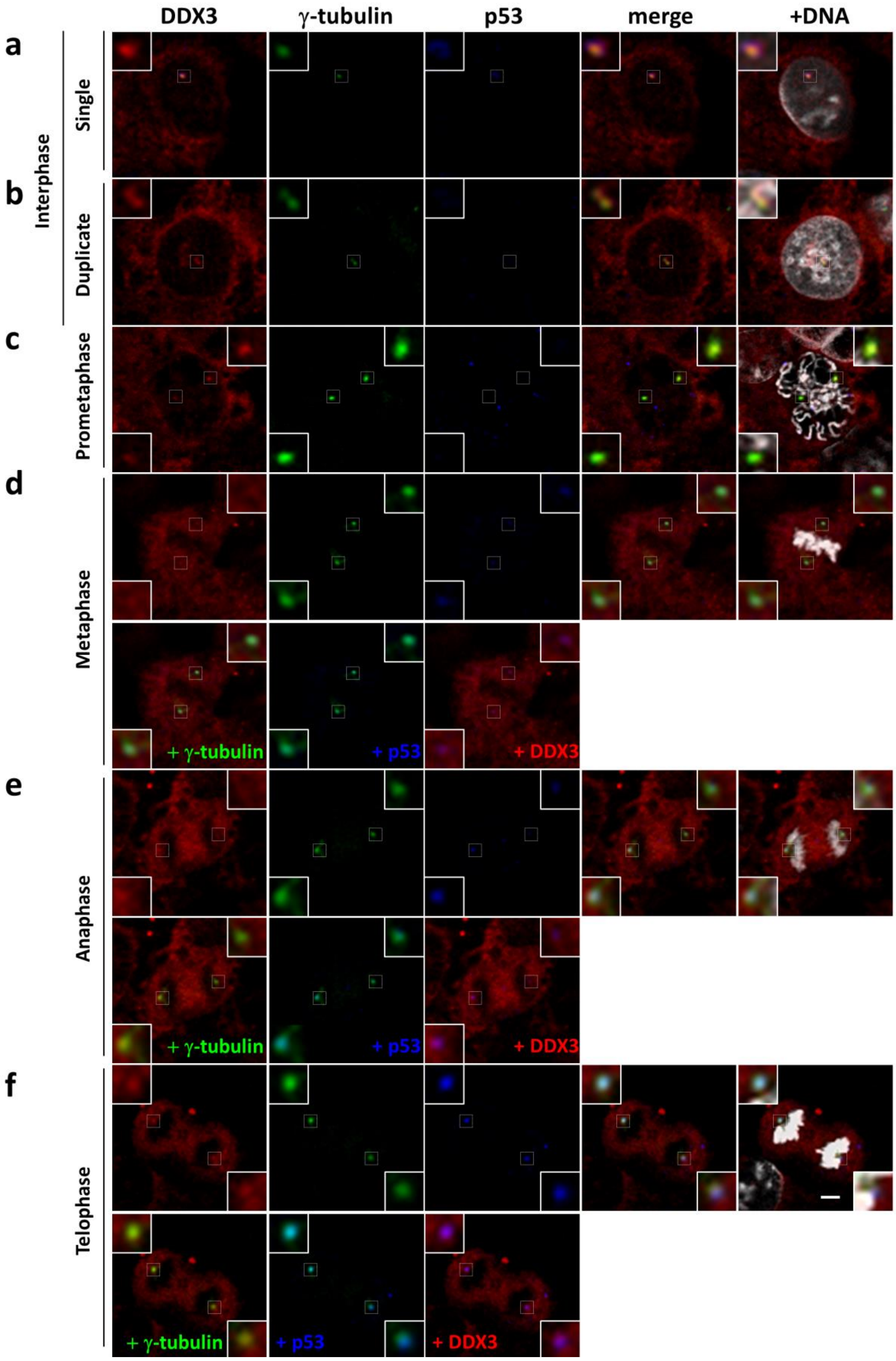

Supplementary Figure S1. Centrosomal targeting of DDX3 throughout the cell cycle and colocalization between p53, DDX3 and centrosomes during anaphase and telophase in U2OS cells. U2OS cells were immunostained with anti- DDX3 (red), anti-γ-tubulin (green), anti-p53 (blue) antibodies and DAPI (gray) during interphase (a-single centrosome; b-duplicated centrosomes), and mitosis (c-prometaphase; d-metaphase; e-anaphase; f-telophase). Insets exhibit higher magnifications of the centrosome. Scale bar = 5 μm.

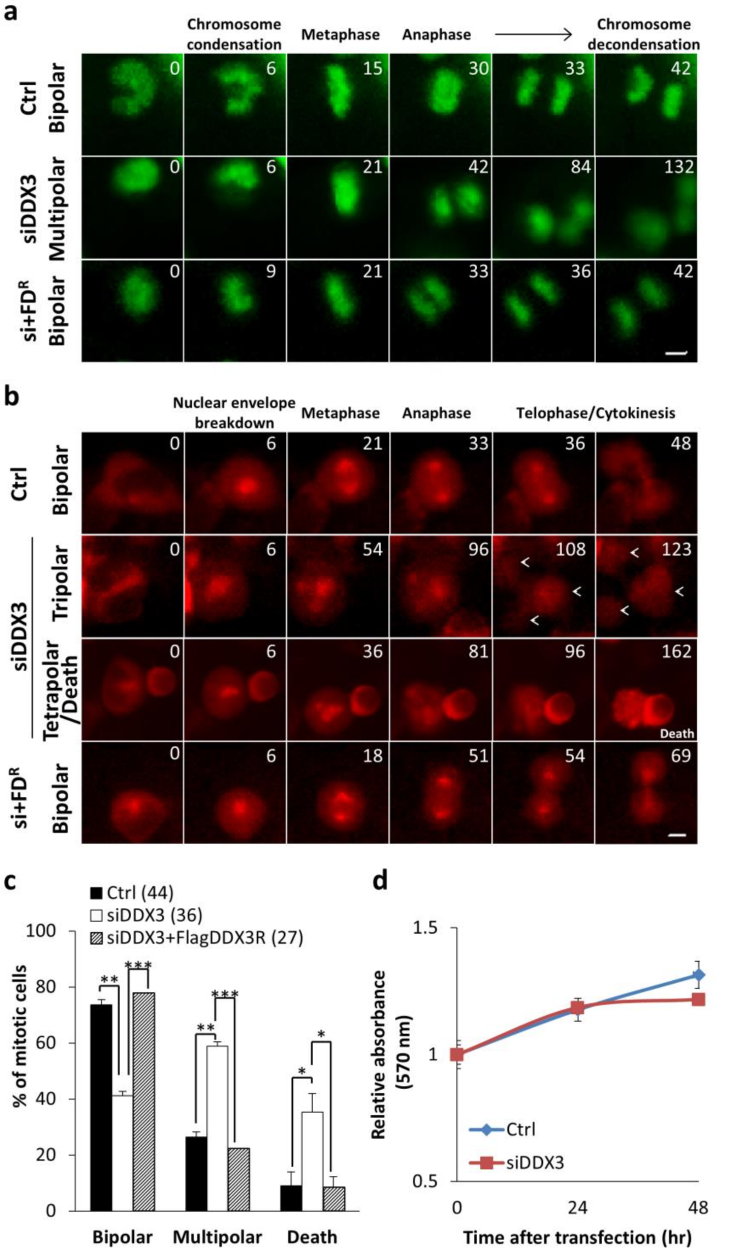

Supplementary Figure S2. Knockdown of DDX3 induced multipolar mitosis and cell death revealed by live cell imaging of the HCT116 cells. a-b. Knockdown of DDX3 prolongs mitosis. Cells were cotransfected with GFP-histone H2B (**a**) or mCherry-α-tubulin (**b**), and control (ctrl), psiDDX3-433 (siDDX3) or psiDDX3-433 coupled with siDDX3-resistant Flag-DDX3 (si+FD^R) to monitor mitotic progression. Number indicates the time in minute. Arrowheads point to the dividing daughter cells. Scale bar = 5 μm. Live cell recordings were performed as described in Supplementary Methods. **c.** Knockdown of DDX3 increases the incidence of multipolar mitosis and cell death. Quantitative analysis of the percentage of bipolar mitosis (comprising normal and pseudo-bipolar mitosis), multipolar mitosis and cell death in the control, siDDX3 and siDDX3+FlagDDX3^R cells of panel b. Data are shown as average value ± S.D. calculated from two independent experiments. *** $P<$ 0.001; **$P<$ 0.01; *$P<$ 0.05. (n), the number of cells analyzed. **d.** Proliferation of vector control (Ctrl) and DDX3-knockdown (siDDX3) cells were analyzed as described in Supplementary Methods.

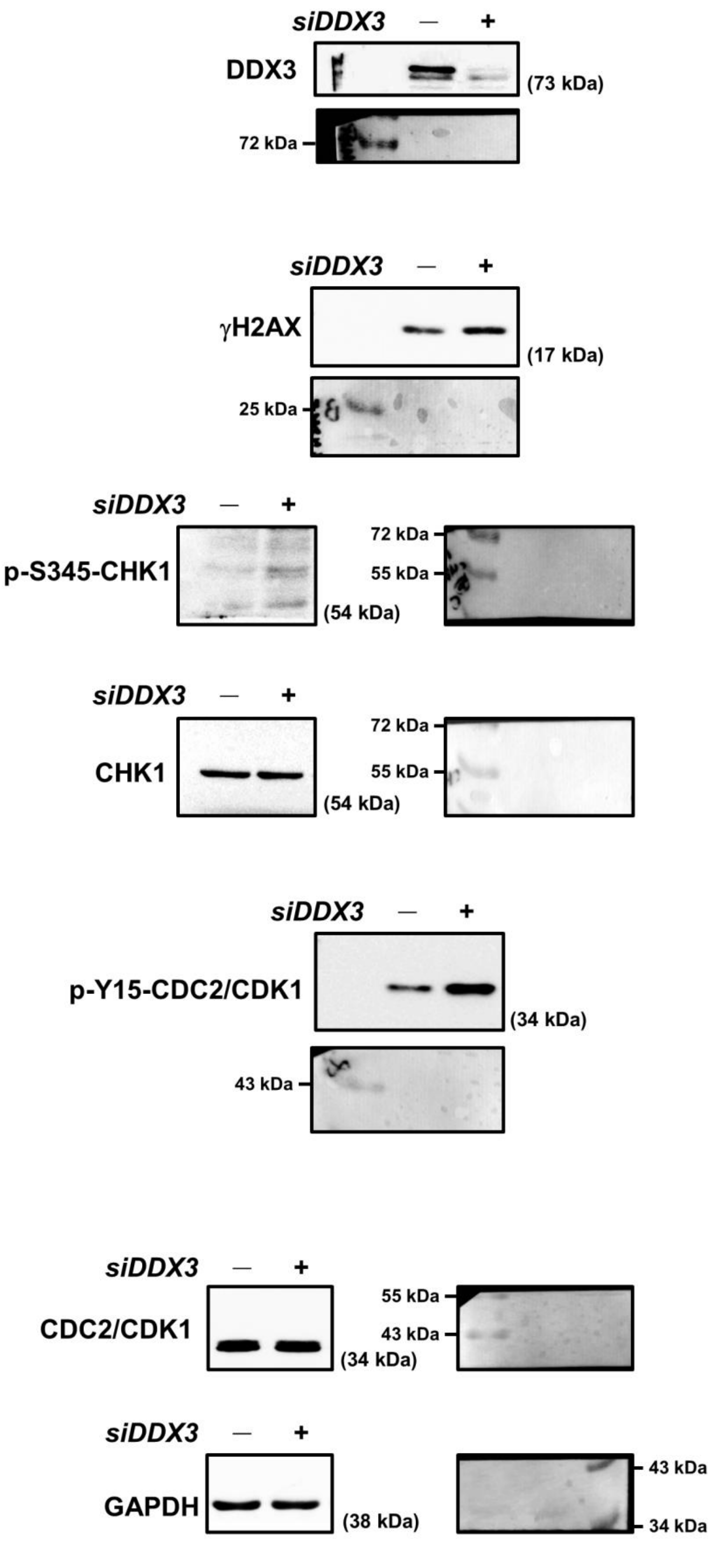

Supplementary Figure S3. Original images of western blots displayed in Figure 2d.

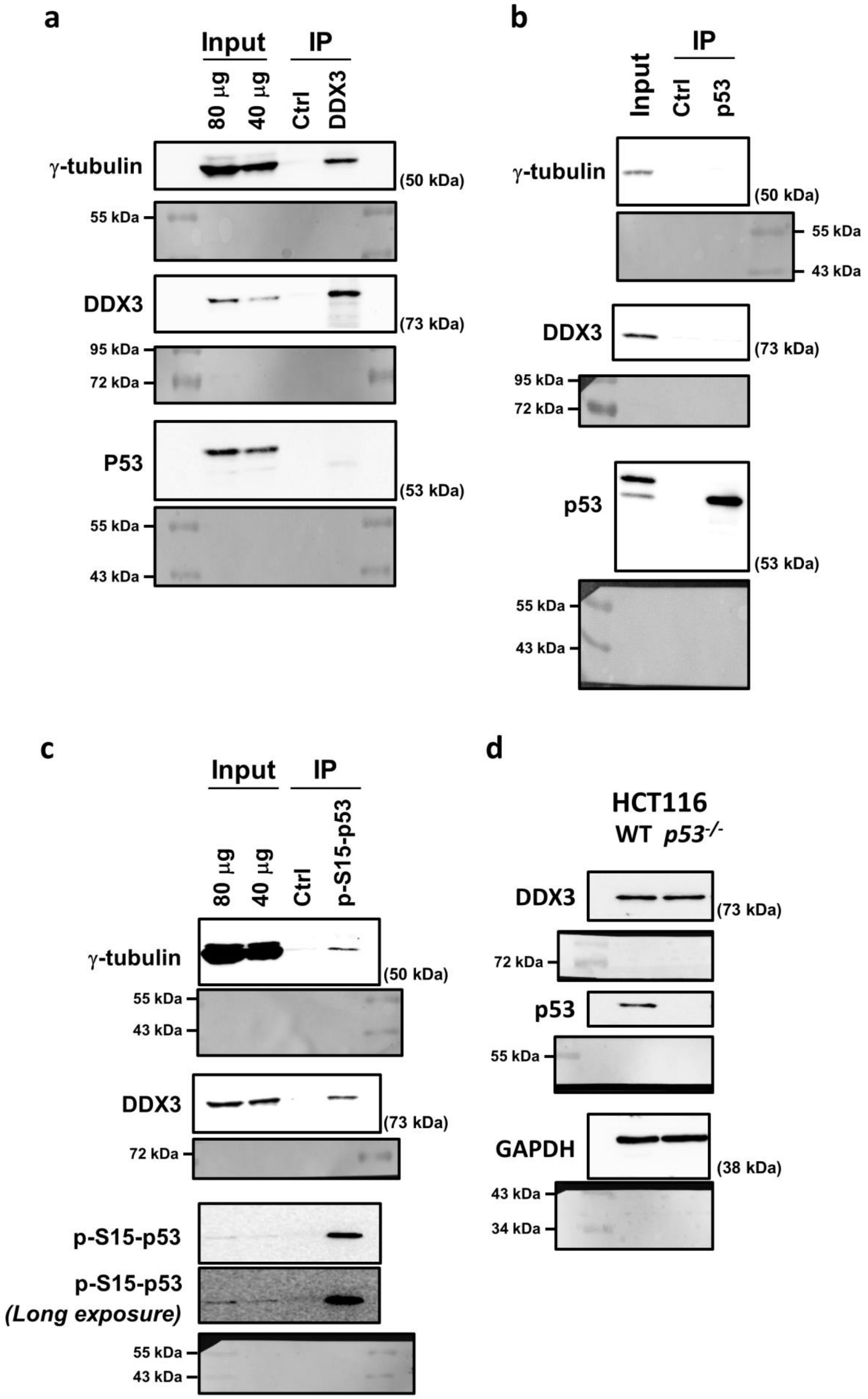

Supplementary Figure S4. Original images of western blots displayed in Figures 5a (a), 5b (b), 5c (c) and 5g (d).

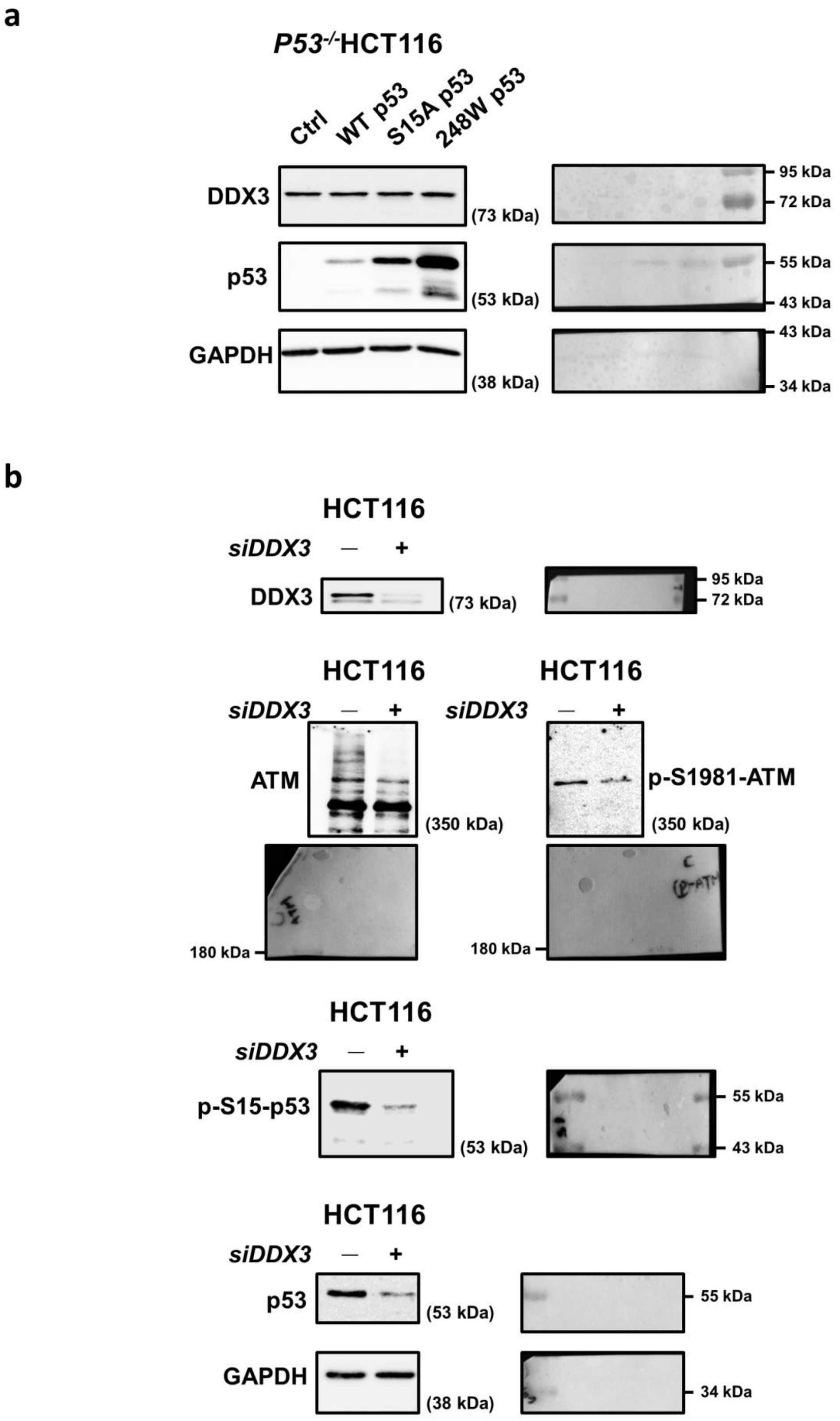

Supplementary Figure S5. Original images of western blots displayed in Figures 6c (a) and 7b (b).

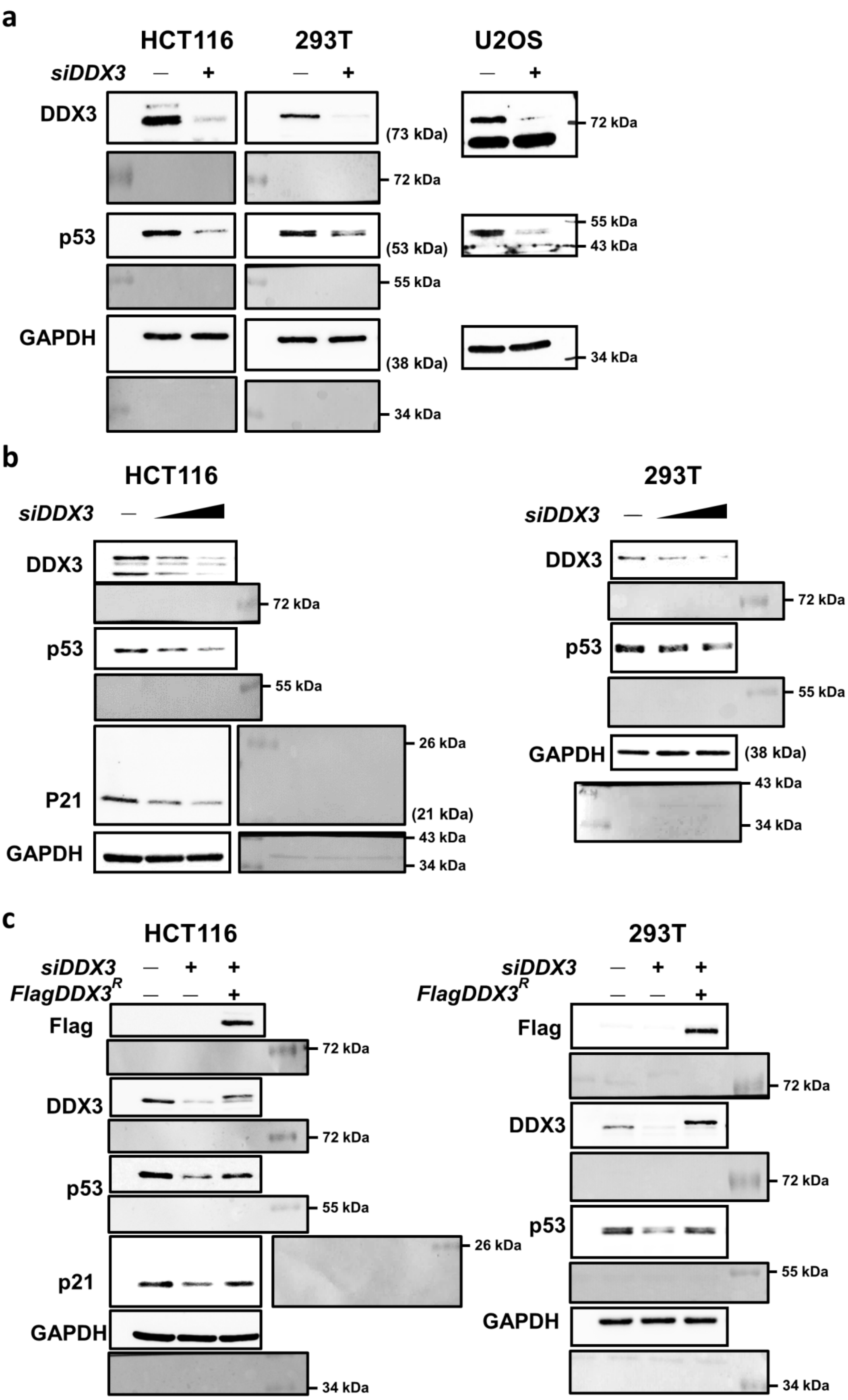

Supplementary Figure S6. Original images of western blots displayed in Figures 8a (a), 8b (b) and 8c (c).

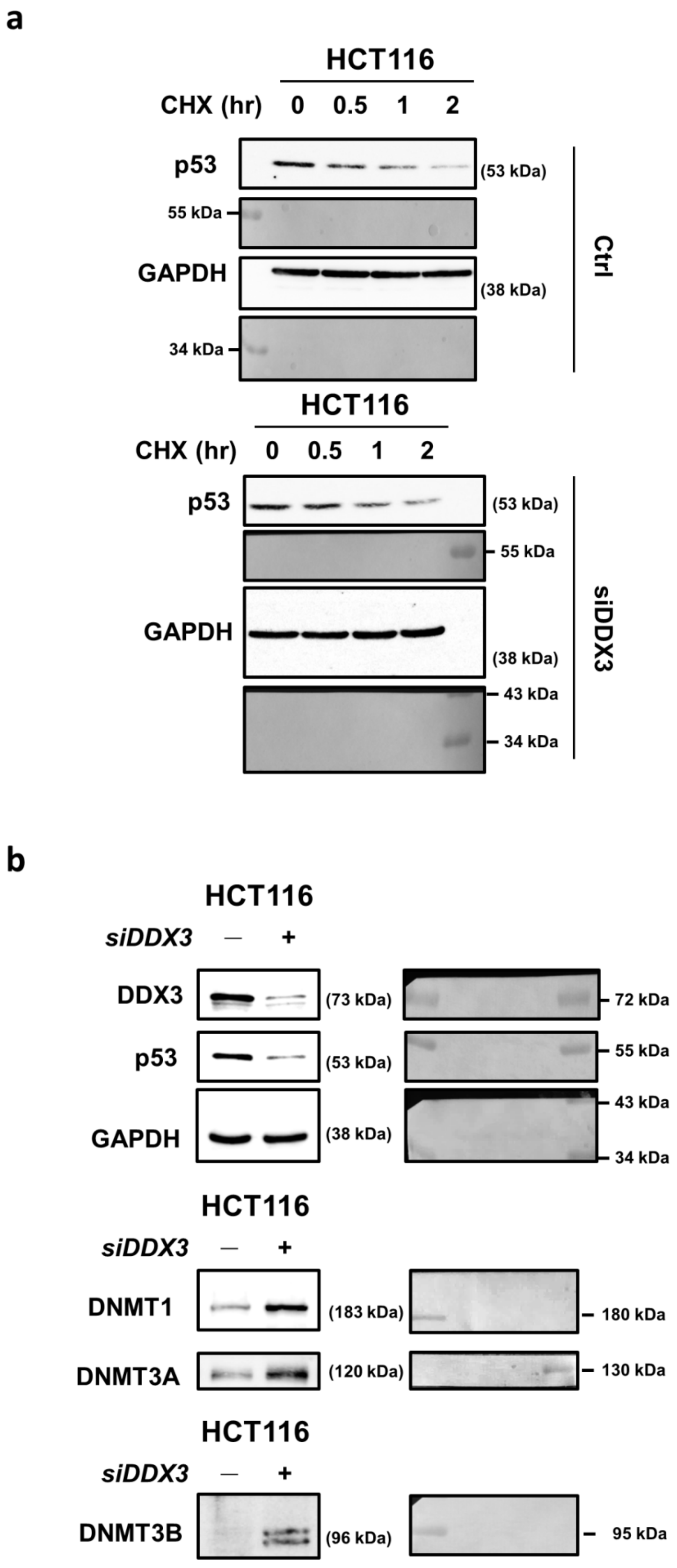

Supplementary Figure S7. Original images of western blots displayed in Figures 8d (a) and 9c (b).

Supplementary Methods

Live cell imaging

Live cell imaging analysis was performed as previously described[1]. For recordings of cell proliferation in a time-lapse manner, HCT116 cells were seeded onto 3.5-cm glass-bottomed dishes (Alpha Plus, Taoyuan, Taiwan), transfected with pcDNA3-mCherry-α-tubulin or GFP-histone H2B and control, psiDDX3-433 or psiDDX3-433 coupled with siDDX3-resistant Flag-DDX3 and maintained in a temperature- and CO_2-controlled chamber. After overnight culture, cells were observed in an inverted microscope (Leica DM IRB) equipped with x20 lenses, and images were captured every 3 minutes.

Proliferation assay

Cells were seeded in a 12-well plate. At 0, 24, 48 hours after transfection with control or psiDDX3-433, cell proliferation was assessed by their ability to transform 3-[4,5-dimethylthiazol-2-yl]-2,5-diphenyltetrazolium bromide (MTT) into purple formazan whose absorbance was determined at 570 nm.

1. Chien, JY *et al.* αTAT1 downregulation induces mitotic catastrophe in HeLa and A549 cells. *Cell Death Disco.* 2, 16006; 10.1038/cddiscovery (2016).
